# The ROCK1 PHC1 domain mediates recruitment to Rho activity zones and contractility signaling

**DOI:** 10.26508/lsa.202603885

**Published:** 2026-10-06

**Authors:** Carolin Gierse, Ingrid R Vetter, Donna Schweizer, Leif Dehmelt

**Affiliations:** 1 Faculty of Chemistry and Chemical Biology, TU Dortmund University , Dortmund, Germany; 2 IMPRS for Living Matter, Max-Planck-Institute of Molecular Physiology, Dortmund, Germany; 3 Department of Mechanistic Cell Biology, Max-Planck-Institute of Molecular Physiology, Dortmund, Germany; 4 Cell Biology, Neurobiology and Biophysics, Department of Biology, Faculty of Science, Utrecht University, Utrecht, The Netherlands; 5 Dortmund Life Science Center (DOLCE), TU Dortmund University, Dortmund, Germany

## Abstract

The spatiotemporal regulation of cell contraction is crucial for numerous biological processes. In particular, local, subcellular contraction pulses in the cell cortex contribute to mechanotransduction and tissue rearrangements during embryonic development. We previously identified a signaling network that generates mechanosensitive contraction pulses in adherent mammalian cells through positive and negative feedback regulation of the small GTPase RhoA. Here, we show that ROCK1 is rapidly recruited to cortical pulsatile Rho activity zones and that efficient recruitment is synergistically mediated by the canonical Rho-binding domain and the C-terminal PHC1 domain. Surprisingly, the isolated ROCK1 PHC1 domain by itself is already sufficient for dynamic recruitment to active Rho signaling zones in cells, and AlphaFold-guided mutagenesis identifies conserved PHC1 residues that are required for this recruitment. Functional investigations show that the PHC1 domain plays an important role in transduction of Rho activity to myosin activation. Together, our data revise models of ROCK1 regulation by identifying a C-terminal PHC1 domain–dependent mechanism that contributes to ROCK1 recruitment and signal transduction during cell contraction pulses.

## Abstract (precis)

Gierse et al show that ROCK1’s PHC1 domain is recruited to elevated cortical Rho activity, that it cooperates with the Rho-binding domain, and that it participates in cell contraction signaling.

## Introduction

Dynamic changes in cell morphology are essential for many biological processes, such as embryonic development, wound healing, or cancer metastasis. These morphological changes are enabled by dynamic rearrangements of the cytoskeleton. In addition to driving cell shape changes, cytoskeletal dynamics are also used by cells to actively probe the mechanical properties of their surroundings (Aguilar-Cuenca et al, 2014). This is achieved by an active probing mechanism, which involves dynamic cell contraction pulses that were shown to transduce mechanical signals more effectively compared with constant contraction (Cui et al, 2015; Graessl et al, 2017; Nalbant & Dehmelt, 2018). The contractile force in these pulses is generated by the action of nonmuscle myosin II, which shifts antiparallel actin filaments against each other near the plasma membrane (Niederman & Pollard, 1975; Svitkina et al, 1995).

**Figure 1.**
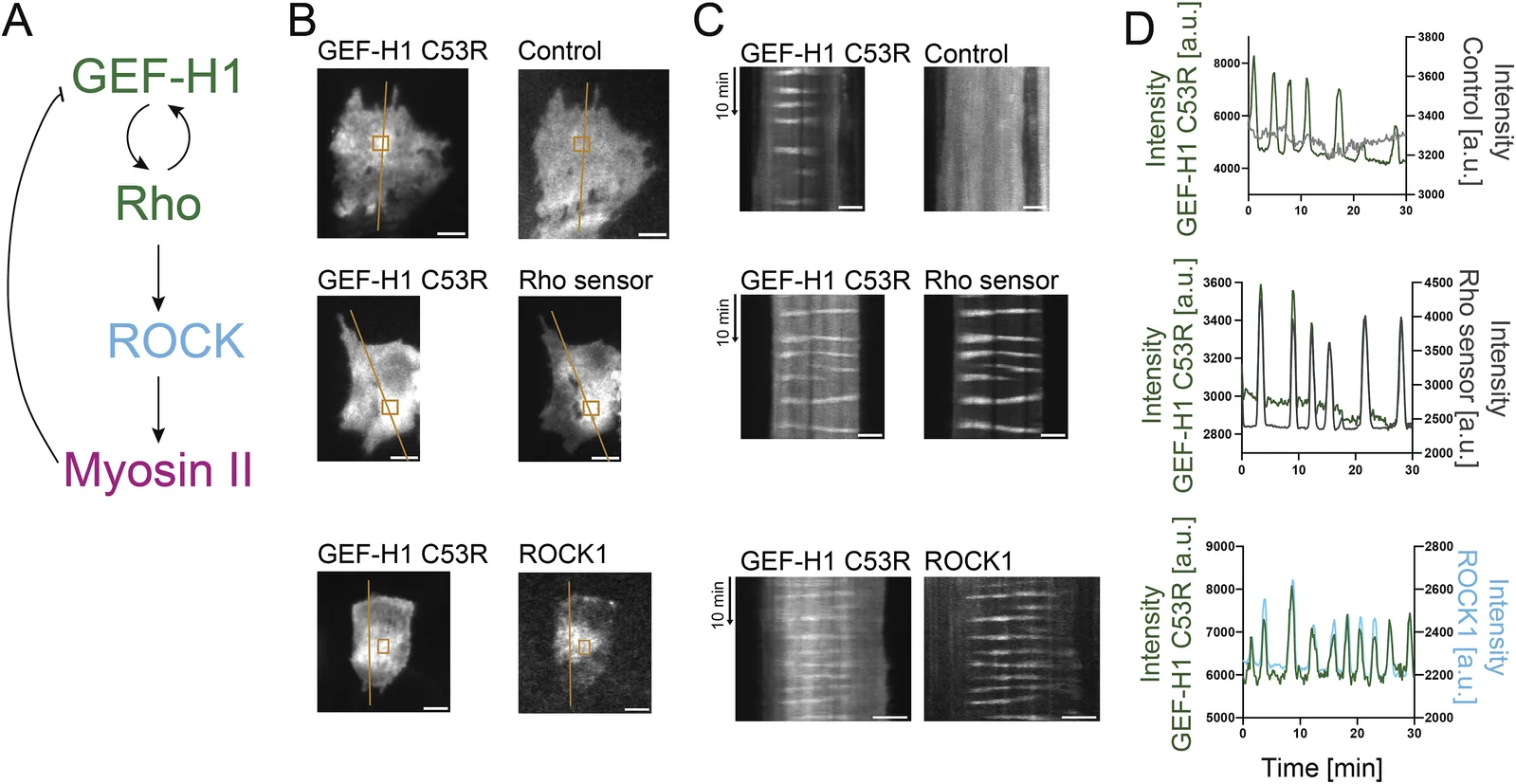
Full-length ROCK1 is dynamically recruited to cortical Rho activity zones. **(A)** Topology of the cell contraction signaling network. GEF-H1 activates the small GTPase Rho, which in turn recruits GEF-H1 to the plasma membrane resulting in positive feedback. Rho activates myosin II via its effector ROCK. Myosin II mediates negative feedback by competing with GEF-H1 (Lee et al, 2010; Graessl et al, 2017; Kamps et al, 2020). **(B, C, D)** Analysis of ROCK1 plasma membrane recruitment dynamics. **(B)** TIRF images of representative cells, in which cell contraction network dynamics were stimulated by the expression of the constitutively active GEF-H1 mutant C53R (mApple-GEF-H1 C53R, top and middle, or mCitrine-GEF-H1 C53R, bottom). Cells additionally co-express one reporter: delCMV-mCitrine as volume marker (top, Video 1B), delCMV-mCitrine-Rhotekin-GBD as Rho activity sensor (middle, Video 1C), or mCherry-ROCK1 full length (bottom, Video 1A). **(C)** Kymographs corresponding to orange lines in (B). **(D)** Kinetics of fluorescence signals in rectangular regions of interest in (B). Scale bars: 10 μm. GBD, GTPase-binding domain. Source data are available for this figure.

Pulsatile cell contraction dynamics are a widespread phenomenon, which is observed in various processes that are associated with the function of individual cells and the development of multicellular structures (Martin et al, 2009; Bement et al, 2015; Graessl et al, 2017; Kim et al, 2018; Michaux et al, 2018; Nalbant & Dehmelt, 2018). In several of these systems, pulsatile dynamics were found to be generated by an activator–inhibitor signal network, which combines rapid positive and slow negative feedback regulation in the cell cortex near the plasma membrane (Bement et al, 2015; Graessl et al, 2017; Michaux et al, 2018). Detailed analyses in adherent mammalian cells revealed that the active form of the small GTPase Rho recruits its activator GEF-H1 (human ARHGEF2, UniProt Q92974) to close a positive feedback loop (Graessl et al, 2017; Kamps et al, 2020), and this fast reaction is coupled to a negative feedback loop, which is mediated by a much slower myosin II–dependent process (Lee et al, 2010; Graessl et al, 2017; Kamps et al, 2020; Fig 1A). The coupling of these processes is dependent on a well-known Rho effector, the Rho kinase ROCK (Fig 1A), via phosphorylation of the myosin II light chain and subsequent activation of myosin II (Amano et al, 1996, 1997; Leung et al, 1996; Mott et al, 1996; Ishizaki et al, 1997; Graessl et al, 2017).

**Figure S1.**
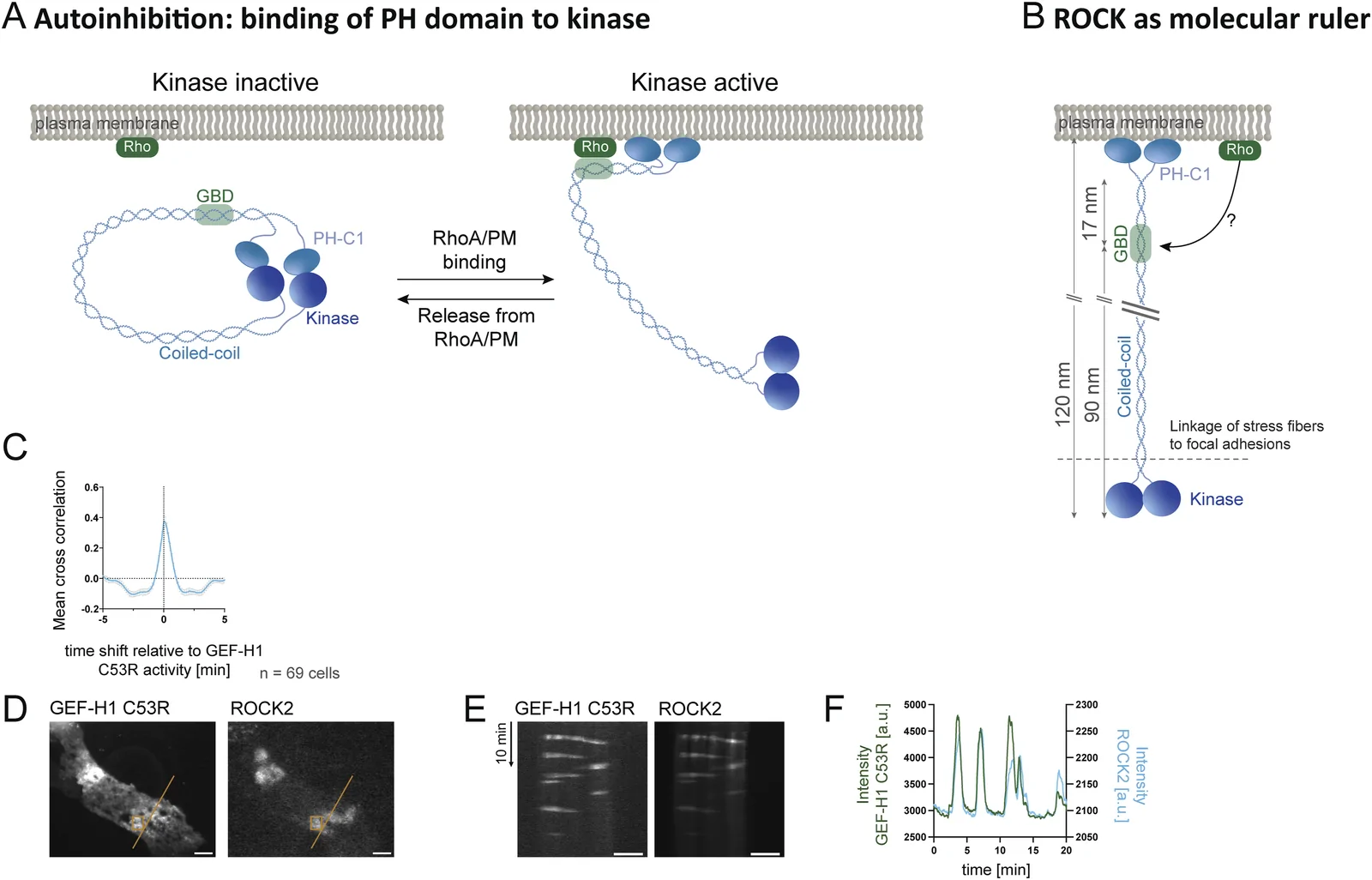
Related to Introduction and Fig 1. **(A)** Classical model of ROCK regulation via autoinhibition, in which the PHC1 domain is thought to inhibit the kinase domain (Leung et al, 1996; Amano et al. 1997, 1998, 1999; Ishizaki et al, 1997; Julian & Olson, 2014; Hartmann et al, 2015). **(B)** More recent model in which the coiled-coil domain positions the kinase domain at a defined distance from the plasma membrane. In this model, ROCK1 is thought to be constitutively bound to the plasma membrane. How RhoA would activate ROCK in this conformation is unclear. **(C)** Cross-correlation analysis of GEF-H1 and ROCK1 (n = 69 cells, four independent experiments; see Materials and Methods for details). **(D, E, F)** Analysis of ROCK2 plasma membrane recruitment dynamics. **(D)** TIRF images of a representative cell co-expressing mCitrine-GEF-H1 C53R and mCherry-ROCK2 full length. **(E)** Kymograph corresponding to the orange lines in (D). **(F)** Kinetics of fluorescence signals in rectangular regions of interest in (D). Error bars represent the standard error of the mean; scale bar = 10 μm. Source data are available for this figure.

Earlier studies suggested a relatively simple mechanism for ROCK activation, in which the C-terminal region of ROCK binds and inhibits the N-terminal kinase domain (Chen et al, 2002), and in which this autoinhibition is released by an interaction between active Rho and the ROCK C terminus (Fig S1A). However, more recent studies based on electron microscopy imaging questioned this idea and proposed that ROCK is always in a fully extended conformation, with its long coiled-coil region of ~90–100 nm acting as a spacer to position the kinase domain at a distance of approx. 120 nm from the plasma membrane (Fig S1B; Truebestein et al, 2015). According to this more recent hypothesis, the N-terminal kinase domain of ROCK would only be able to reach and activate myosin in the dense cell cortex near the plasma membrane in this fully extended conformation. However, it remains unclear how active Rho is able to activate ROCK, as all previously described Rho-binding sites are located within the coiled-coil domain, far away from the plasma membrane in the fully extended ROCK conformation. Furthermore, these more recent studies failed to confirm the previously proposed interaction between ROCK and active Rho, thus leaving a gap in our understanding how ROCK and its downstream targets can be activated.

Here, we investigated how ROCK1 (human ROCK1, UniProt Q13464) is coupled to Rho activity during pulsatile cell contraction dynamics. We found that full-length ROCK1 is recruited to pulsatile Rho activity zones within seconds, well before the recruitment and activation of myosin II reaches its maximum. Surprisingly, for this rapid recruitment of ROCK1, none of its previously described Rho interaction domains were essential. Instead, the C-terminal PHC1 tandem domain was sufficient for highly effective plasma membrane recruitment to cortical Rho activity zones. Based on predictions using AlphaFold, we identified four conserved amino acids that significantly contribute to this recruitment. Optogenetic manipulations of ROCK1 suggest that the PHC1 domain and the AlphaFold-predicted amino acids play additional roles beyond plasma membrane recruitment. We conclude that the monomeric PHC1 (mPHC1) domain contributes to ROCK1 recruitment to cortical Rho activity zones and that an additional function of this domain apart from localizing to the actin cortex is necessary for ROCK1 to carry out its function to activate myosin II.

## Results

### ROCK1 is dynamically recruited to cell contraction pulses in adherent mammalian cells

In previous studies, we identified and characterized a signaling network in mammalian, adherent cells, which can generate spontaneous cell contraction pulses at the plasma membrane (Fig 1A; Graessl et al, 2017; Kamps et al, 2020). In these studies, we used total internal reflection fluorescence (TIRF) microscopy to detect the plasma membrane translocation of a fluorescent-labeled Rho effector domain (Benink & Bement, 2005) to measure local Rho activity dynamics within individual pulses (Graessl et al, 2017) and we combined this with measurements of plasma membrane translocation of Rho-activating GEFs and of Rho downstream targets, such as F-actin, FHOD1, and myosin (Graessl et al, 2017; Kamps et al, 2020). These studies revealed that Rho and its activator GEF-H1 can rapidly amplify each other with a minimal temporal time lag of less than 3 s and that the recruitment and activation of downstream targets are significantly delayed (Graessl et al, 2017). In particular, maximal myosin II recruitment at the plasma membrane—as detected by TIRF microscopy—occurred about 40 s after maximal Rho activation (Graessl et al, 2017). This recruitment was strongly correlated with the formation of centripetal contractile flows, which shows that myosin II is not only recruited to pulsatile Rho activity zones at the plasma membrane but also locally activated at these pulses (Graessl et al, 2017). Here, we used a similar strategy to investigate the dynamics of the Rho effector ROCK1 within individual cell contraction pulses (Fig 1B–D and Video 1). Analogous to our previous studies, we simply express a constitutively active, microtubule binding–deficient GEF-H1 C53R mutant to stimulate the generation of local, subcellular activity pulses of the cell contraction signal network, and thereby enhance the sensitivity of our analyses (Krendel et al, 2002; Graessl et al, 2017; Kamps et al, 2020). As shown in middle panels of Fig 1B–D, we confirmed our previous observation (Graessl et al, 2017) that the spatiotemporal plasma membrane recruitment dynamics of fluorescent-labeled GEF-H1 C53R and of the Rho activity sensor are identical. For the following studies, we therefore use GEF-H1 C53R both as a stimulator of cell contraction pulses and as a sensor to report spatiotemporal Rho activity dynamics.

Video 1 Related to Fig 1. **(A)** GEF-H1 C53R expression stimulates cell contraction network dynamics to form pulse- and wave-like activity patterns. Time-lapse TIRF videos of mCitrine-GEF-H1 C53R and mCherry in a representative U2OS cell. **(B)** Rho activity strongly correlates with GEF-H1 C53R–stimulated cell contraction signal network activity in space and time. Time-lapse TIRF videos of mApple-GEF-H1 C53R and the Rho activity sensor mCitrine-Rhotekin-GBD in a representative U2OS cell. **(C)** ROCK1 strongly correlates with GEF-H1 C53R–stimulated cell contraction signal network activity at the plasma membrane. Time-lapse TIRF videos of mCitrine-GEF-H1 C53R and mCherry-ROCK1 full length in a representative U2OS cell. Imaging started 42 h after plating on collagen-coated glass-bottom dishes. Images were collected with a frame rate of 7.5/min. Scale bars, 10 μm. GBD, GTPase-binding domain. Download video

Using this approach, we investigated the spatiotemporal relationship between human full-length mCherry-ROCK1 and its activator Rho (Fig 1B–D, bottom, Video 1A). These experiments clearly show that ROCK1 plasma membrane recruitment closely follows GEF-H1 C53R in space and time throughout the whole-cell attachment area with minimal temporal delay. Detailed cross-correlation analyses show that ROCK1 is maximally recruited just 3.12 ± 0.56 s (mean ± SEM) after GEF-H1 C53R (Fig S1C). Analogous experiments with a ROCK2 (human ROCK2, UniProt O75116) construct showed similar results (Fig S1D–F).

### The ROCK1 PHC1 domain is sufficient for recruitment of ROCK1 to Rho activity pulses

**Figure 2.**
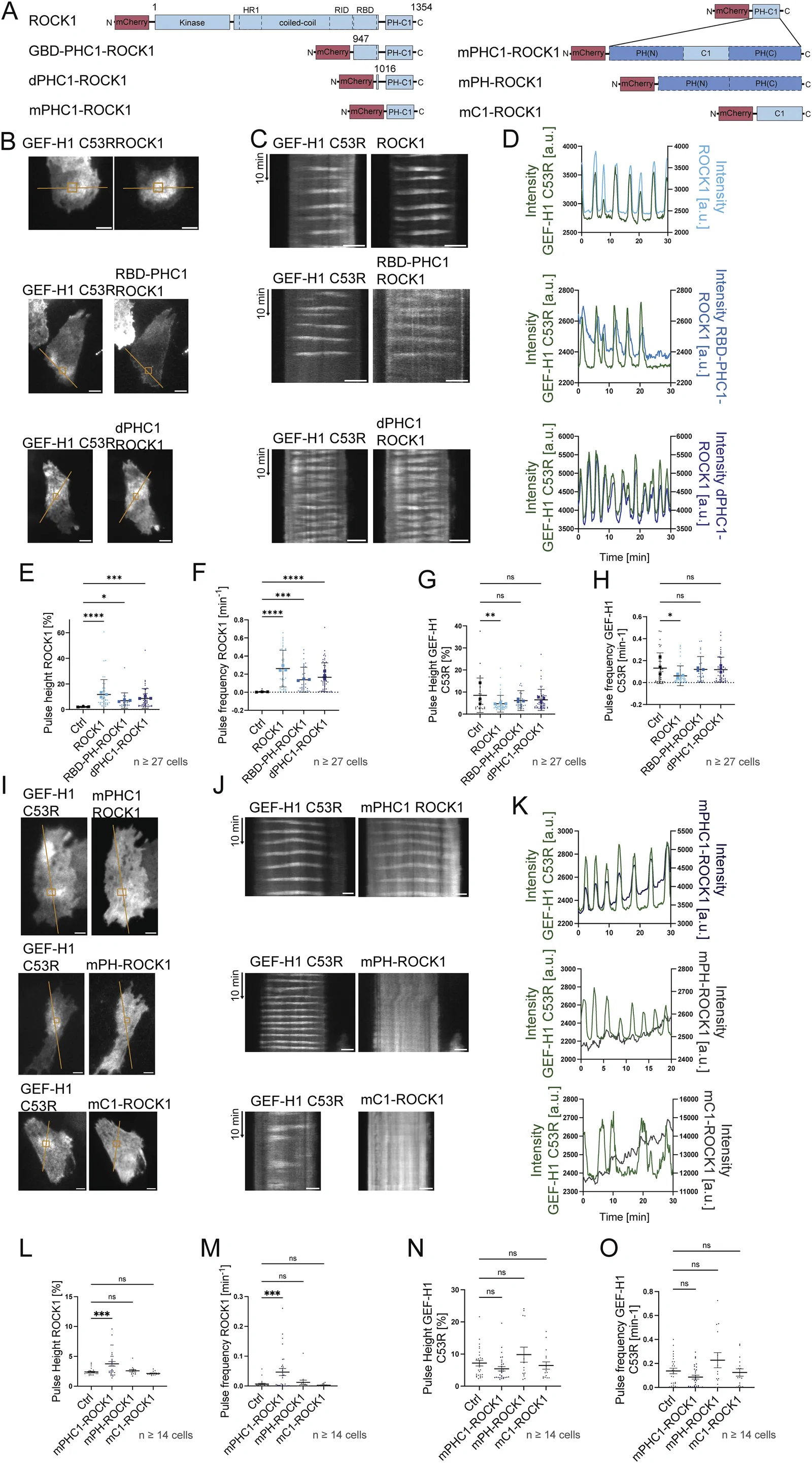
The mPHC1 tandem domain of ROCK1 is sufficient for recruitment to Rho activity zones. **(A)** Schematic representation of ROCK1 constructs used in this study (HR1: homology region 1; RID: Rho-interacting domain; RBD: Rho-binding domain; PH: Pleckstrin homology; C1: phorbol ester/diacylglycerol-binding domain; the d and m prefixes indicate presumed monomeric or dimeric constructs, based on the absence or presence of the coiled-coil region). **(B, C, D, E, F)** Analysis of plasma membrane recruitment dynamics of N-terminal ROCK1 truncation mutants. **(B)** TIRF images of representative cells co-expressing mCitrine-GEF-H1 C53R and mCherry-ROCK1 full length (top, Video 2A), mCherry-RBD-PHC1-ROCK1 (middle, Video 2B), or mCherry-dPHC1-ROCK1 (bottom, Video 2C). **(C)** Kymographs corresponding to the orange lines in (B). **(D)** Kinetics of fluorescence signals in rectangular regions of interest in (B). **(E, F)** Quantitative analysis of plasma membrane recruitment dynamics of mCherry-ROCK1 constructs shown in (B, C, D), compared with the control (empty parental mCherry vector). Means of individual repeats are shown as bold squares. **(G, H)** Quantitative analysis of signal network dynamics by measurements of mCitrine-GEF-H1 plasma membrane recruitment in the same experiment as shown in (E, F). **(I, J, K, L, M)** Analysis of plasma membrane recruitment dynamics of the ROCK1 mPH and mC1 domains, as well as the mPHC1 tandem domain. Means of individual repeats are shown as bold squares. **(I)** TIRF images of representative cells co-expressing mCitrine-GEF-H1 C53R and mCherry-mPHC1-ROCK1 (top, Video 3A), mCherry-mPH-ROCK1 (middle, Video 3B), or mCherry-mC1-ROCK1 (bottom, Video 3C). **(J)** Kymographs corresponding to the orange lines in (I). **(K)** Kinetics of fluorescence signals in rectangular regions of interest in (I). **(L, M)** Quantitative analysis of plasma membrane recruitment dynamics of mCherry-ROCK1 constructs shown in (I, J, K), compared with the control (empty parental mCherry vector). **(N, O)** Quantitative analysis of signal network dynamics by measurements of mCitrine-GEF-H1 plasma membrane recruitment in the same experiment as shown in (L, M). Error bars represent the standard error of the mean from three independent experiments each. *P < 0.05, **P < 0.01, ***P < 0.001, ****P < 0.0001; one-way ANOVA with Dunnett’s posttest, comparison of means of each condition with the mean of the “Ctrl” condition; scale bars: 10 μm. Source data are available for this figure.

A recent study (Truebestein et al, 2015) has cast doubt on a direct interaction between ROCK1 and active Rho, in particular via the previously characterized Rho-binding domain (RBD, residues 994–1009) within its coiled coil, raising the question how the tight spatiotemporal cross-correlation between ROCK1 and GEF-H1 (Fig 1B–D) could be explained (Truebestein et al, 2015). In addition to this canonical RBD region, two additional Rho-binding sites were previously identified in ROCK (Fig 2A): the homology region 1 (residues 420–550) and the Rho-interacting domain (residues 842–931) (Blumenstein & Ahmadian, 2004). These additional potential binding sites are also localized in the coiled-coil domain (Truebestein et al, 2015). At the very C terminus, ROCK has a pleckstrin homology (PH) domain with a cysteine-rich (C1) domain inserted within the PH domain (PHC1 tandem domain in the following), and this domain was shown to bind the plasma membrane via a cluster of positive charges (Wen et al, 2008). In other proteins, including Lbc-type GEFs and Raf kinase, PH and C1 domains were found to specifically interact with the active form of small GTPases (Mott et al, 1996; Graessl et al, 2017; Kamps et al, 2020). We therefore hypothesized that this ROCK1 domain might not only interact with the plasma membrane, but might also more specifically contribute to its recruitment to Rho activity pulses.

To address this question, we generated N-terminally truncated human ROCK1 constructs. The first set of constructs that we investigated contained either both the originally identified canonical RBD (Blumenstein & Ahmadian, 2004) and the PHC1 tandem domain (RBD-PHC1-ROCK1, residues 947–1354) or only the PHC1 tandem domain together with a minimal coiled-coil region to enable its dimerization (dPHC1-ROCK1, residues 1016–1354) (Fig 2A). Thus, RBD-PHC1-ROCK1 contains the previously characterized canonical RhoA-binding domain, whereas dPHC1-ROCK1 does not contain any previously reported RhoA-binding site.

We then measured the dynamic recruitment of these constructs to cell contraction pulses in U2OS cells, in which the signal network dynamics were stimulated by GEF-H1 C53R co-expression (Graessl et al, 2017; Kamps et al, 2020; Fig 2B–D and Video 2). As expected, RBD-PHC1-ROCK1 plasma membrane recruitment closely correlates with maximal Rho activity indicated by GEF-H1 C53R signals. Interestingly, dPHC1-ROCK1, which contains none of the previously postulated RBDs (Blumenstein & Ahmadian, 2004), equally colocalized with GEF-H1 dynamics. This shows that none of the previously postulated RBDs are strictly required to recruit ROCK1 to sites of increased GEF-H1/Rho activity (Fig 2 B–D and Video 2). Furthermore, quantitative analysis of ROCK1 pulse dynamics (Graessl et al, 2017) confirms that both truncations exhibit pulsatile plasma membrane recruitment dynamics that are statistically indistinguishable (Fig 2E and F).

Video 2 Related to Fig 2. **(A)** ROCK1 is strongly recruited to GEF-H1 C53R–stimulated cell contraction signal network activity at the plasma membrane. Time-lapse TIRF videos of mCitrine-GEF-H1 C53R and mCherry-ROCK1 full length in a representative U2OS cell. **(B)** N-terminal truncated RBD-PH-ROCK1 is strongly recruited to GEF-H1 C53R–stimulated cell contraction signal network activity at the plasma membrane. Time-lapse TIRF videos of mCitrine-GEF-H1 C53R and mCherry-RBD-PH-ROCK1 in a representative U2OS cell. **(C)** N-terminal truncated PHC1-ROCK1 is strongly recruited to GEF-H1 C53R–stimulated cell contraction signal network activity at the plasma membrane. PHC1-ROCK1 does not contain any of the previously reported Rho-binding regions of ROCK1. Time-lapse TIRF videos of mCitrine-GEF-H1 C53R and mCherry-dPHC1-ROCK1 in a representative U2OS cell. Imaging started 42 h after plating on collagen-coated glass-bottom dishes. Images were collected with a frame rate of 7.5/min. Scale bars, 10 μm. Download video

We next investigated whether these ROCK1 constructs affect cell contraction signal network dynamics. To evaluate this, we measured the dynamics of the GEF-H1 signals in the presence of these ROCK1 constructs. These measurements show that ROCK1 wild type had an inhibitory effect, whereas RBD-PHC1-ROCK and dPHC1-ROCK1 did not influence dynamics, concerning neither GEF-H1 peak height nor GEF-H1 pulse frequency (Fig 2G and H and Video 2). This was initially surprising, as previous studies suggested an inhibitory, dominant-negative effect of the PHC1 domain of ROCK on cell contraction (Leung et al, 1996; Amano et al, 1997; Ishizaki et al, 1997). This observation shows that the two truncated constructs do not influence oscillatory cell contraction dynamics and that instead, increasing the expression level of the wild-type molecule, which includes the N-terminal kinase domain, inhibits cell contraction signal network dynamics.

To narrow down the region sufficient for recruitment to Rho activity zones, we removed the small, dimerizing coiled coil that was still present in the dPHC1-ROCK1 construct, to obtain a mPHC1 domain (residues 1119–1354, Fig 2A). As shown in Fig 2I–K (top, Video 3A), the plasma membrane recruitment of the mPHC1 domain still clearly and closely correlates with GEF-H1 signals in space and time.

Video 3 Related to Fig 2. **(A)** The mPHC1 tandem domain of ROCK1 (mPHC1-ROCK1) is recruited to GEF-H1 C53R–stimulated cell contraction signal network activity at the plasma membrane. mPHC1-ROCK1 does not contain any of the previously identified GTPase-binding domains of ROCK1 nor any known dimerization motifs. Time-lapse TIRF videos of mCitrine-GEF-H1 C53R and mCherry-mPHC1-ROCK1 in a representative U2OS cell. **(B)** mPH-ROCK1 (PH part of PHC1 domain) is not efficiently recruited to GEF-H1 C53R–stimulated cell contraction signal network activity at the plasma membrane. Time-lapse TIRF videos of mCitrine-GEF-H1 C53R and mCherry-mPH-ROCK1 in a representative U2OS cell. **(C)** mC1-ROCK1 (C1 part of PHC1 domain) is not efficiently recruited to GEF-H1 C53R–stimulated cell contraction signal network activity at the plasma membrane. Time-lapse TIRF videos of mCitrine-GEF-H1 C53R and mCherry-mPH-ROCK1 in a representative U2OS cell. Imaging started 42 h after plating on collagen-coated glass-bottom dishes. Images were collected with a frame rate of 7.5/min. Scale bars, 10 μm. Download video

Because both the PH and the C1 domains of other proteins were previously shown to bind small GTPases (Mott et al, 1996; Kamps et al, 2020; Powis et al, 2023), we further dissected the tandem domain to obtain the individual, monomeric PH (mPH-ROCK1, residues 1119–1228 and 1282–1354, connected via a flexible synthetic linker) and C1 (mC1-ROCK1, residues 1229–1281) domains (Fig 2A). The mC1-ROCK1 domain did not exhibit any detectable signal changes in relation to GEF-H1 (Fig 2I–K, bottom, Video 3C), and the mPH-ROCK1 was only very weakly enriched at local GEF-H1 signals (Fig 2I–K, middle, Video 3B). The automated peak analysis confirmed these observations: mC1-ROCK1 did not show an increase in the ROCK1 pulse height, and mPH-ROCK1 showed a very small trend, which was not significantly different compared with the empty control construct (Fig 2L and M). This shows that both domains together are essential for efficient plasma membrane recruitment of the C-terminal region of ROCK1 to Rho activity zones.

In addition, we did not observe any reduction in network activity upon expression of either mPHC1-, mPH-, nor mC1-ROCK1, further supporting our observation that the PH domain itself has no dominant-negative effect on pulsatile cell contraction dynamics (Fig 2N and O).

### Identification of amino acids that are critical for the mPHC1 domain recruitment to pulsatile Rho activity zones

**Figure 3.**
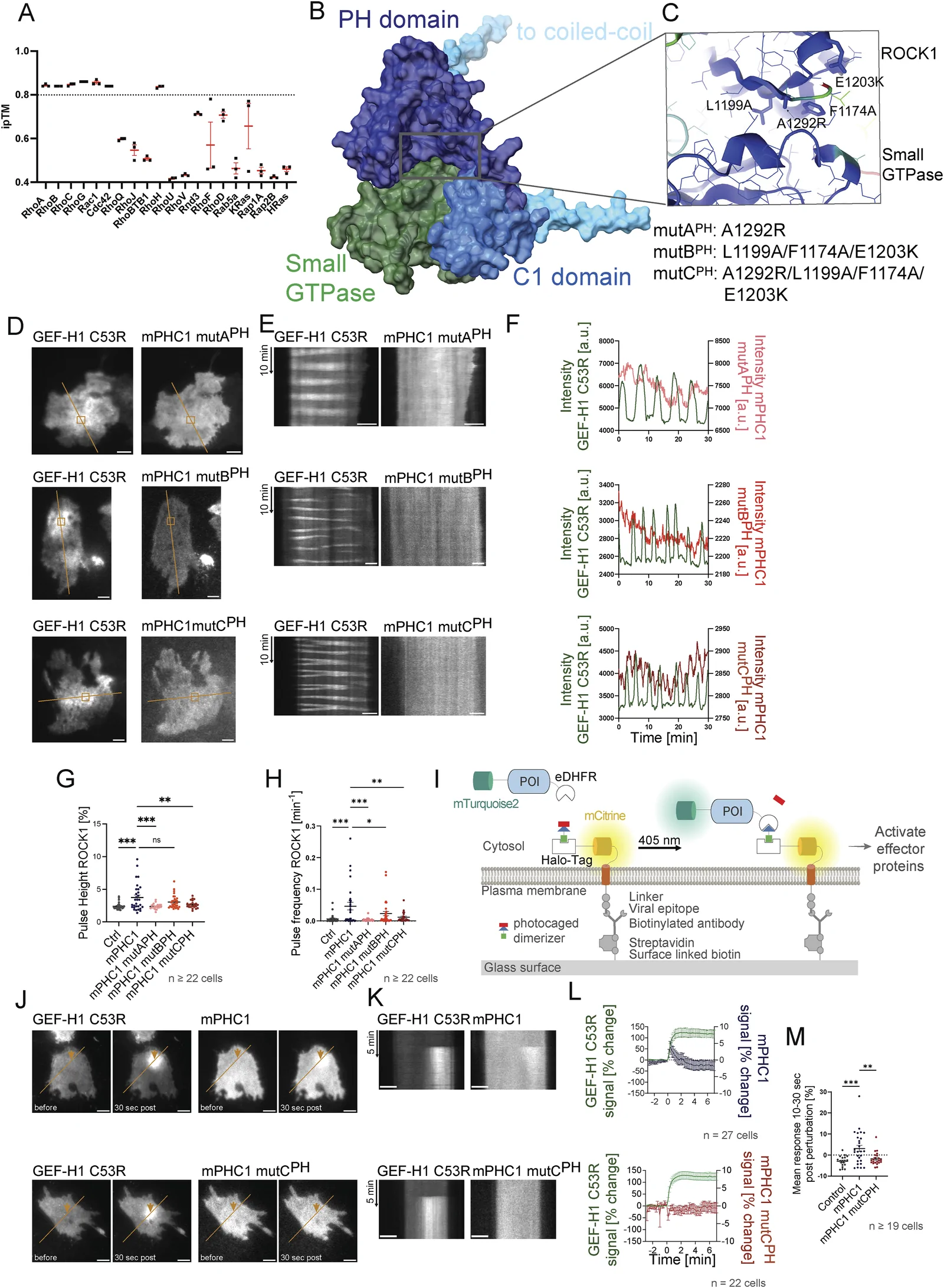
AlphaFold-guided identification of PHC1 residues required for recruitment to Rho activity zones. **(A, B, C)** AlphaFold prediction of complex formation between PHC1 and small GTPases. **(A)** ipTM score of various GTP-bound small GTPases in complex with the PHC1 tandem domain obtained via AlphaFold predictions. The dotted line indicates an empirical confidence threshold often used to prioritize predicted interfaces. Several GTPases, in particular of the Rho subfamily, were predicted to form potential complexes with the ROCK1 mPHC1 domain. **(B)** Structure of the predicted complex (blue: PHC1 tandem domain; green: small GTPase). The complex of PHC1 with RhoA is shown as a representative example. **(C)** Magnified view of the predicted binding interface between the PH domain of ROCK1 and the small GTPase. Amino acids that contribute most to the predicted binding interface via Protein Interfaces, Surfaces, and Assemblies are indicated. **(D, E, F, G, H)** Analysis of plasma membrane recruitment dynamics of ROCK1 mPHC1 domain point mutants. **(D)** TIRF images of representative cells co-expressing mCitrine-GEF-H1 and mCherry-mPHC1 mutA^PH^ (top, Video 4A), mCherry-mPHC1 mutB^PH^ (middle, Video 4B), or mCherry-mPHC1 mutC^PH^ (bottom, Video 4C). **(E)** Kymographs corresponding to lines in (D). **(F)** Kinetics of fluorescence signals in rectangular regions of interest in (D). **(G, H)** Quantitative analysis of plasma membrane recruitment dynamics of mCherry-ROCK1 constructs shown in (D, E, F), compared with the control (empty parental mCherry vector). **(I, J, K, L, M)** Analysis of plasma membrane recruitment of ROCK1 mPHC1 domain point mutants to local, light-induced stimulations of the cell contraction signal network. **(I)** Schematic of the Molecular Activity Painting approach used to introduce local perturbations. In this method, a single pulse of near-UV light (405 nm) is used to trigger the interaction between eDHFR and a plasma membrane–anchored HaloTag. This leads to the plasma membrane recruitment and enrichment of a POI fused to eDHFR. The enriched POI can introduce a local perturbation of signal network activity at the plasma membrane. TIRF microscopy is used to measure the kinetics of the perturbation and of the downstream response. **(J)** TIRF images of the mTurquoise2-eDHFR-GEF-H1 C53R perturbation construct (left) and mCherry-mPHC1 (upper right, Video 5A) or mCherry-mPHC1 mutC^PH^ (bottom right, Video 5B), before and 30 s after initiation of a perturbation at a local spot at the plasma membrane. Orange arrows indicate the position of photodimerizer uncaging. **(K)** Kymographs corresponding to lines in (J). **(L)** Kinetics of mean fluorescence signals measured at the perturbation spot. **(M)** Quantitative analysis of the ROCK1 construct response 10–30 s after light-induced GEF-H1 perturbation. Error bars represent the standard error of the mean from three independent predictions or experiments each. *P < 0.05, **P < 0.01, ***P < 0.001; one-way ANOVA with Dunnett’s posttest, comparison of means of each condition with the mean of the “mPHC1” condition; scale bars: 10 μm. POI, protein of interest. Source data are available for this figure.

**Figure S2.**
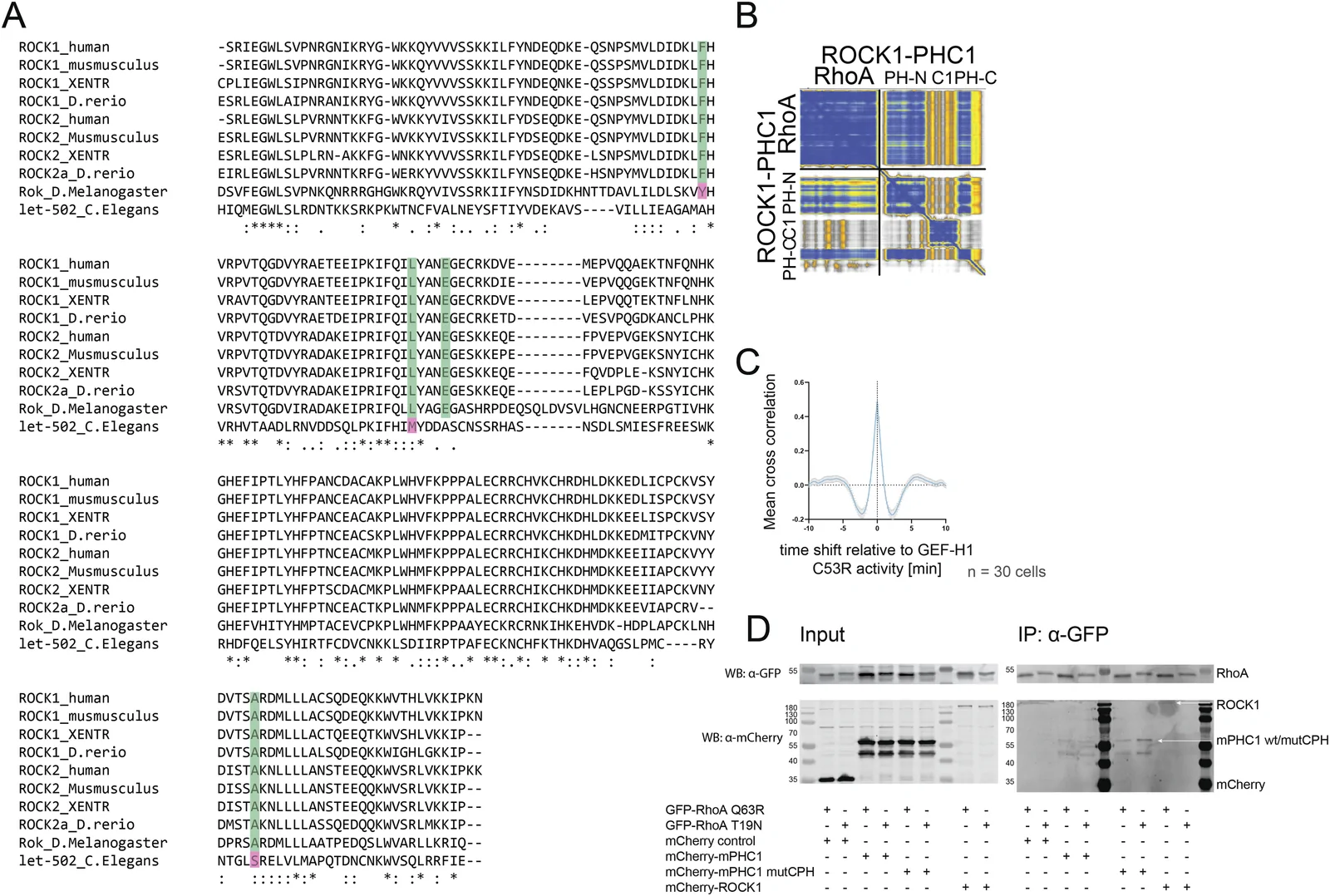
Related to Fig 3. **(A)** Protein sequence alignment of PH domains derived from ROCK1, ROCK2 and their homologs across species using the Clustal 2.1 multiple sequence alignment web tool. **(B)** PAE plot for the predicted PHC1/small GTPase complex. The complex of PHC1 with RhoA is shown as a representative example. **(C)** Cross-correlation analysis of GEF-H1 and mPHC1-ROCK1 (n = 30 cells). Error bars represent the standard error of the mean from three independent experiments. **(D)** Representative pulldown of different ROCK1 variants from HEK cells with RhoA Q63L or RhoA T19N as bait. Molecular weight markers were loaded into lanes 1, 8, 15, and 20. The band with lower molecular weight in the mPHC1-containing lanes is presumably a degradation product. PAE, Predicted Aligned Error. See source data for the uncropped blots of all repetitions. Source data are available for this figure.

To further characterize how mPHC1-ROCK1 is recruited to cell contraction network activity, we employed AlphaFold to investigate whether the PHC1 domain contains a surface that could, in principle, associate with small Rho-family GTPases. Indeed, AlphaFold-predicted complexes between mPHC1 and several GTP-bound Rho-family GTPases with high confidence (Fig 3A). Compared with small GTPases of the Ras, Rap, and Rab families, the interface predicted template modeling (ipTM) score for several members of the Rho subfamily, including RhoA, RhoB, RhoC, RhoG, Rac1, Cdc42, and RhoH, was consistently higher than the empirical confidence threshold of 0.8, which is often used to prioritize predicted interfaces (Fig 3A). Closer inspection of the predicted interaction surfaces between the PHC1 domain and the small GTPases showed that they were very similar. Fig 3B and C shows the predicted PHC1/RhoA complex as a representative example for the various potential small GTPase interaction partners of the PHC1 domain, and Fig S2B shows the corresponding PAE plot.

In contrast to other PH or C1 domains, which can selectively bind signaling lipids such as phosphoinositides or diacylglycerol, respectively, the tandem PHC1 domain of ROCK1 lacks the signatures for binding these molecules (Wen et al, 2008). Instead, the ROCK PHC1 domain has unconventional lipid membrane–binding properties that are based on a cluster of positive charges (Wen et al, 2008). Interestingly, these positive charges are located next to the predicted interface and oriented in the same direction as the plasma membrane anchor of the small GTPase (Fig S4C and D). Thus, the positive charges of the PHC1 domain together with the GTPase plasma membrane anchor might act synergistically to support plasma membrane recruitment in cells.

To investigate the functional relevance of the AlphaFold-predicted interface for ROCK1 function, a Protein Interfaces, Surfaces, and Assemblies analysis was performed (Collaborative Computational Project, Number 4, 1994). This analysis predicted that the four amino acids A1292, F1174, L1199, and E1203 contribute most to the AlphaFold-predicted interface. Interestingly, all of these amino acids are highly conserved in both ROCK1 and ROCK2 in vertebrates and invertebrates (Fig S2A). A notable exception is the more distantly related ROCK ortholog let-502 in *Caenorhabditis elegans*, for which none of the four amino acids are conserved. Nevertheless, AlphaFold predicts a complex between the *C. elegans* ROCK ortholog and either human or *C. elegans* Rho (Fig S5; see Materials and Methods for details). Interestingly, the amino acids predicted to be important for these interactions are distinct and in addition involve the C1 domain (Fig S5, R1113 and Y1114 of C1 contact the small GTPase). In the PH domain, M1029 in *C. elegans* corresponds to L1199 in human and is conserved as a hydrophobic residue. The conformation of the RhoA switch II domain differs in *C. elegans*, which can explain the different conservation of critical amino acid residues. Taken together, these analyses suggest that the PHC1 surface implicated by these predictions might also contribute to the recruitment of ROCK2 and analogous proteins in other species.

These four amino acids in the PHC1 domain were then mutated to disrupt the predicted PHC1 interaction surface based on electrostatic and steric properties, with minimal impact on the structure of the individual domains. The following mutations were predicted to have the strongest effect: A1292R, F1174A, L1199A, and E1203K (Fig 3C; mPHC1 mutA^PH^: A1292R; mPHC1 mutB^PH^: F1174A, L1199A, E1203K; mPHC1 mutC^PH^: A1292R, F1174A, L1199A, E1203K).

Experimental investigations showed that two independent sets of mPHC1 domain mutations (the single mutant mutA^PH^ and the triple mutant mutB^PH^) display significantly reduced recruitment to GEF-H1/Rho activity signal dynamics: we measured a strong reduction in the recruitment of a single amino acid mutant (mPHC1 mutA^PH^: A1292R; Fig 3D–F, top, Video 4A), and the independent, nonoverlapping triple mutant (mPHC1 mutB^PH^: F1174A, L1199A, E1203K, Video 4B) also showed significantly reduced signals (Fig 3 D–F, middle). Combining the triple mutant with the A1292R mutation (mutC^PH^) further reduced the signals (Fig 3D–F, bottom, Video 4C). Quantitative peak analysis confirmed that all three mutants show strongly reduced peak height and frequency compared with the nonmutated mPHC1-ROCK1 domain that served as a control (Fig 3G and H).

Video 4 Related to Fig 3. **(A)** Mutated mPHC1 tandem domain of ROCK1 (mPHC1-ROCK1 mutA^PH^) with the A1292R point mutation is not efficiently recruited to GEF-H1 C53R–stimulated cell contraction signal network activity at the plasma membrane. Time-lapse TIRF videos of mCitrine-GEF-H1 C53R and mCherry-mPHC1-ROCK1 mutA^PH^ in a representative U2OS cell. **(B)** Mutated mPHC1 tandem domain of ROCK1 (mPHC1-ROCK1 mutB^PH^) with the L1199A/F1174A/E1203K point mutations is not efficiently recruited to GEF-H1 C53R–stimulated cell contraction signal network activity at the plasma membrane. Time-lapse TIRF videos of mCitrine-GEF-H1 C53R and mCherry-mPHC1-ROCK1 mutB^PH^ in a representative U2OS cell. **(C)** Mutated mPHC1 tandem domain of ROCK1 (mPHC1-ROCK1 mutC^PH^) with the L1199A/F1174A/E1203K/A1292R point mutations is not recruited to GEF-H1 C53R–stimulated cell contraction signal network activity at the plasma membrane. Time-lapse TIRF videos of mCitrine-GEF-H1 C53R and mCherry-mPHC1-ROCK1 mutC^PH^ in a representative U2OS cell. Imaging started 42 h after plating on collagen-coated glass-bottom dishes. Images were collected with a frame rate of 7.5/min. Scale bars, 10 μm. Download video

The peak analysis clearly shows that mPHC1 plasma membrane recruitment coincides with GEF-H1/Rho activity signal network activity, and a more detailed cross-correlation analysis confirms a strong spatiotemporal correlation between these signals (Fig S2C). To directly test whether mPHC1 plasma membrane recruitment is indeed caused by increased GEF-H1/Rho activity signal network activity, we employed Molecular Activity Painting, a rapid chemo-optogenetic approach to introduce local perturbations in signal networks, by recruiting a protein of interest to the plasma membrane (Chen et al, 2017; Kamps et al, 2020; Fig 3I). Here, the constitutively active RhoGEF GEF-H1 C53R is the protein of interest, which is fused to a fluorophore and the heterodimerization domain *Escherichia coli* dihydrofolate reductase (eDHFR). A HaloTag acts as a second heterodimerization domain. This domain is immobilized at the plasma membrane by fusion to an artificial receptor, which is bound to surface-linked antibodies. A photocaged small molecule dimerizer binds covalently to the HaloTag, and uncaging by a single pulse of light triggers the plasma membrane recruitment of RhoGEF GEF-H1 C53R-eDHFR. At the plasma membrane, GEF-H1 C53R can activate endogenous Rho (Kamps et al, 2020; Kowalczyk et al, 2022), as well as Rho effectors.

Upon recruitment of GEF-H1 C53R to the plasma membrane, we observed a significant increase in wild-type mPHC1-ROCK signals. This confirms a causal link between GEF-H1–induced cell contraction signal network activity and mPHC1-ROCK1 plasma membrane recruitment (Fig 3J and K and Video 5A). Furthermore, we performed the same experiment with the quadruple mPHC1 mutant (mutC^PH^) and did not detect any local enrichment of this construct (Fig 3J and K and Video 5B).

Video 5 Related to Fig 3. **(A)** Rapid chemo-optogenetic plasma membrane recruitment of GEF-H1 C53R leads to recruitment of the mPHC1 domain of ROCK1 at a distinct site at the plasma membrane. TIRF video microscopy of a representative U2OS cell, expressing the perturbation construct mTurquoise2-eDHFR-GEF-H1C53R (left), the response construct mCherry-mPHC1-ROCK1 (right), and the artificial receptor mCitrine-VSVG HaloTag-PARC. **(B)** Rapid chemo-optogenetic plasma membrane recruitment of GEF-H1 C53R does not lead to plasma membrane recruitment of the mutated monomeric mPHC1 mutC^PH^ domain of ROCK1 with the L1199A/F1174A/E1203K/A1292R point mutations. TIRF video microscopy of a representative U2OS cell, expressing the perturbation construct mTurquoise2-eDHFR-GEF-H1C53R (left), the response construct mCherry-mPHC1mutC^PH^-ROCK1 (right), and the artificial receptor mCitrine-VSVG HaloTag-PARC. A focused light pulse at 405 nm was applied at timepoint 0 to target the perturbation construct to a small region of the plasma membrane. Images were collected with a frame rate of 6/min. Scale bars, 10 μm. Download video

Interestingly, the wild-type mPHC1 response to the GEF-H1 C53R perturbation is only transient (Fig 3L). This further supports the hypothesis that PHC1 plasma membrane recruitment is linked to the comparably transient Rho activity dynamics, which was previously described to be triggered by local optogenetic GEF-H1 C53R perturbation (Kamps et al, 2020; Kowalczyk et al, 2022).

As PHC1 recruitment dynamics and mutational analysis suggested a close link to Rho activity in cells, we tested whether the isolated PHC1 domain and the active form of the best-studied isoform RhoA interact with each other. We therefore performed pull-down experiments using proteins expressed in mammalian HEK293T cells. In these pull-down assays, we were able to confirm specific binding of mCherry-ROCK1 full length to active RhoA Q63L and no binding to inactive RhoA T19N, whereas the negative control (mCherry) did not interact with Rho in either activity state (Fig S2D and SourceDataFigS2). Furthermore, RhoA Q63L was reproducibly able to pull down mCherry-mPHC1; however, an equally strong signal was also detected using inactive RhoA T19N, and also with mCherry-mPHC1 mutC^PH^ (included in one experimental repetition) (Fig S2D).

Thus, under the soluble pull-down conditions used here, the isolated PHC1 domain did not exhibit RhoA activity-state specificity or dependence on the AlphaFold-predicted interface. This assay therefore did not provide evidence for a direct high-affinity PHC1-RhoA interaction, but it does not exclude interactions with other Rho-family GTPases like the highly related RhoB and RhoC isoforms, or a weaker interaction with RhoA that could be strengthened by cooperative effects at the plasma membrane that might depend on GTPase membrane anchoring, protein–lipid interactions, or additional cellular cofactors that are absent in the pull-down conditions.

### Both the PHC1 domain and the RBD of ROCK1 contribute to its recruitment to Rho activity zones

**Figure 4.**
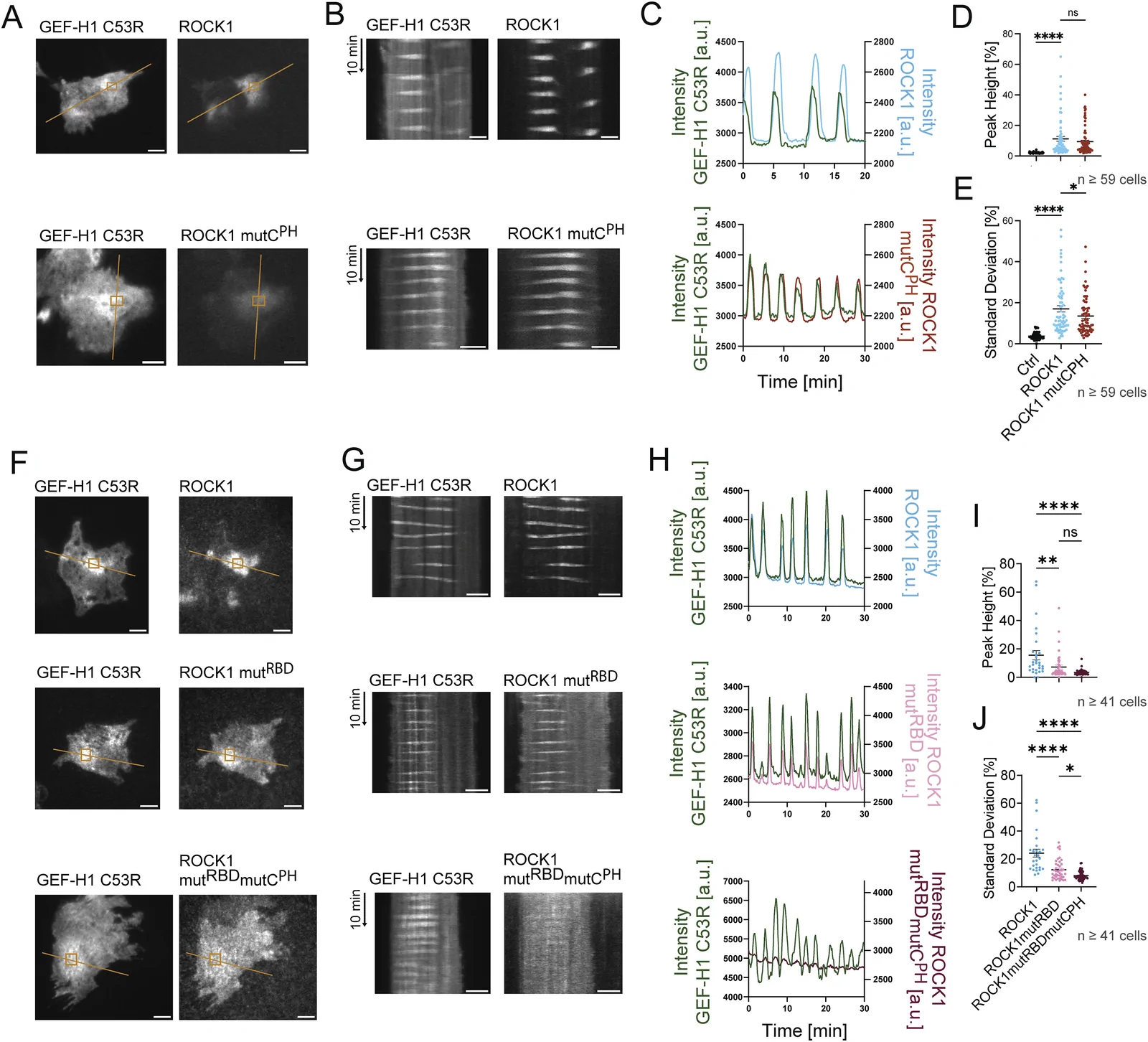
The PHC1 domain and canonical RBD cooperate in ROCK1 recruitment to Rho activity zones. **(A, B, C, D, E)** Analysis of plasma membrane recruitment dynamics of ROCK1 full-length point mutants. **(A)** TIRF images of representative cells co-expressing mCitrine-GEF-H1 and mCherry-ROCK1 (top, Video 6A) or mCherry-ROCK1 mutC^PH^ (bottom, Video 6B) (A). **(B)** Kymographs corresponding to lines in (A). **(C)** Kinetics of fluorescence signals in rectangular regions of interest in (A). **(D, E)** Quantitative analysis of plasma membrane recruitment dynamics of mCherry-ROCK1 signals shown in (A, B, C). **(D)** Quantification of pulse height (ROCK1 versus ROCK1mutC^PH^: *P* = 0.3791). **(E)** Quantification of the local standard deviation over time (ROCK1 versus ROCK1mutC^PH^: *P* = 0.0465; four independent experiments). An empty parental mCherry construct served as a control in (D, E). **(F, G, H, I, J)** Analysis of plasma membrane recruitment dynamics of ROCK1 full-length point mutants. **(F)** TIRF images of representative cells co-expressing mCitrine-GEF-H1 and mCherry-ROCK1 (top, Video 8A), mCherry-ROCK1 mut^RBD^ (middle, Video 8B), or mCherry-ROCK1 mut^RBD^ mutC^PH^ (bottom, Video 8C). **(G)** Kymographs corresponding to lines in (F). **(H)** Kinetics of fluorescence signals in rectangular regions of interest in (F). **(I, J)** Quantitative analysis of plasma membrane recruitment dynamics of mCherry-ROCK1 signals shown in (F, G, H). **(I)** Quantification of pulse height. **(J)** Quantification of the local standard deviation over time (three independent experiments). *P < 0.05, **P < 0.01, ****P < 0.0001; one-way ANOVA with (D, E) Dunnett’s posttest, comparison of means of each condition with the mean of the “ROCK1” condition or Tukey’s posttest (I, J); scale bars: 10 μm. Source data are available for this figure.

We next investigated the functional relevance of the PHC1 domain. First, we introduced the four point mutations into the PHC1 domain of human full-length ROCK1 to examine their role in recruitment to cortical Rho activity zones. As shown in Fig 4A–C (bottom), we found that ROCK1 full-length mutC^PH^ still exhibits pulsatile behavior that closely correlates with GEF-H1 in space and time, presumably because of the presence of other previously identified Rho-interacting regions, including the canonical RBD (Fujisawa et al, 1996; Leung et al, 1996; Blumenstein & Ahmadian, 2004; Dvorsky et al, 2004; Tu et al, 2011). This correlation was similar compared with wild-type ROCK1 (Fig 4A–C, top, and Video 6). However, analysis of the peak height showed a trend that this mutant was less effectively recruited to GEF-H1 at the plasma membrane (*P* = 0.3791, Fig 4D). Notably, the more sensitive measurement of the standard deviation of fluorescence intensity signals showed significantly reduced dynamics of the ROCK1 full-length mutC^PH^ compared with nonmutated ROCK1 full length at the plasma membrane, further supporting the idea that the PHC1 domain contributes to ROCK1 recruitment (Fig 4E).

Video 6 Related to Fig 4. **(A)** Full-length ROCK1 is strongly recruited to GEF-H1 C53R–stimulated cell contraction signal network activity at the plasma membrane. Time-lapse TIRF videos of mCitrine-GEF-H1 C53R and mCherry-ROCK1 in a representative U2OS cell. **(B)** Reduced plasma membrane recruitment of mutated full-length ROCK1 mutC^PH^ with the L1199A/F1174A/E1203K/A1292R point mutations to GEF-H1 C53R–stimulated cell contraction signal network activity at the plasma membrane. Time-lapse TIRF videos of mCitrine-GEF-H1 C53R and mCherry-ROCK1mutC^PH^ in a representative U2OS cell. Imaging started 42 h after plating on collagen-coated glass-bottom dishes. Images were collected with a frame rate of 7.5/min. Scale bars, 10 μm. Download video

**Figure S3.**
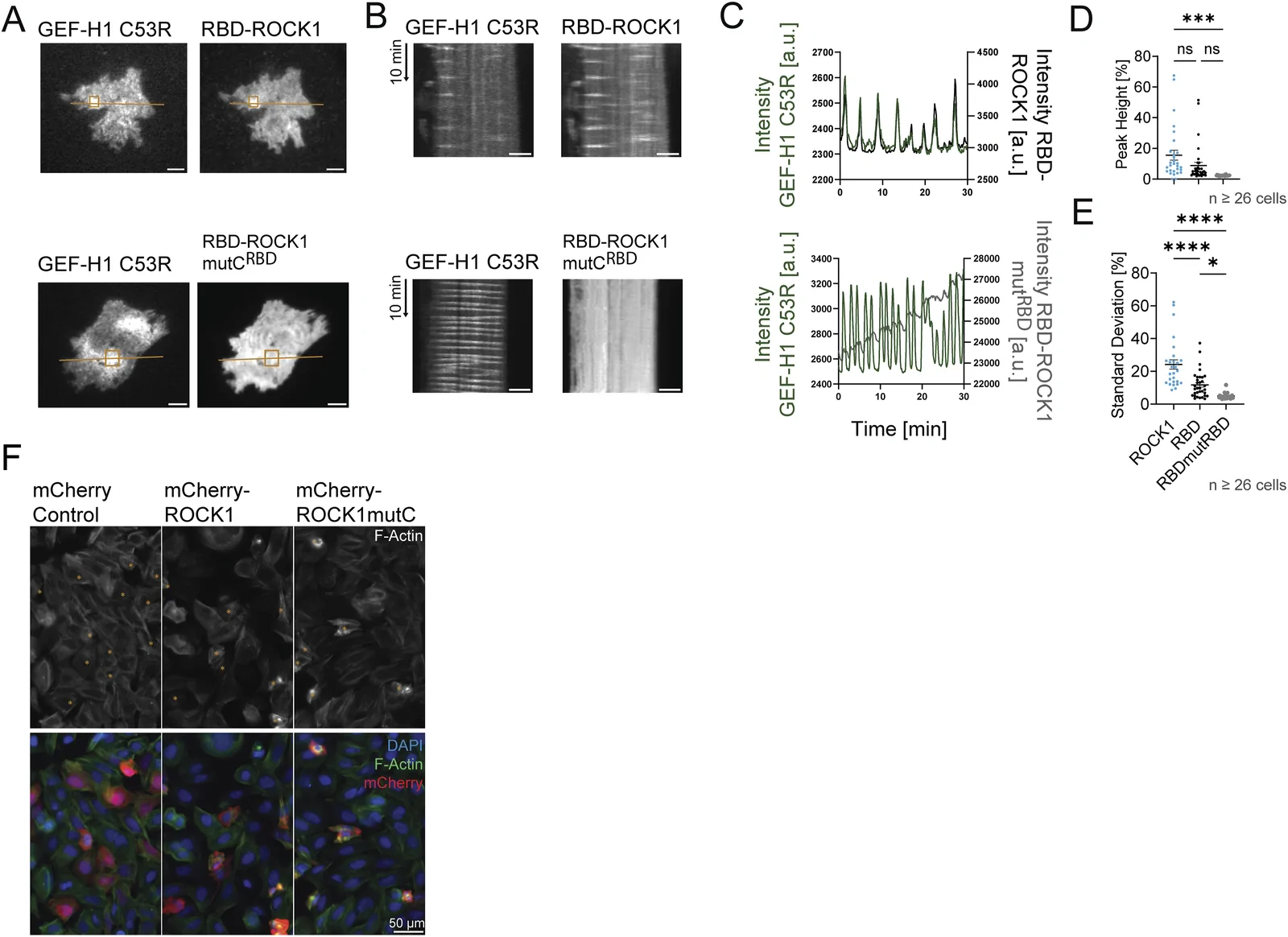
Related to Figs 4 and 5. **(A)** TIRF images of representative cells co-expressing mCitrine-GEF-H1 and mCherry-RBD-ROCK1 (top, Video 7A) or mCherry-RBD-ROCK1 mut^RBD^ (bottom, Video 7B). **(B)** Kymographs corresponding to lines in (A). **(C)** Kinetics of fluorescence signals in rectangular regions of interest in (A). **(D, E)** Quantitative analysis of plasma membrane recruitment dynamics of mCherry-RBD-ROCK1 signals shown in (A, B, C). The control data (ROCK1 full length) are the same as shown in Fig 4, included here again to allow comparison with the treatment group. **(D)** Quantification of pulse height. **(E)** Quantification of the local standard deviation over time (three independent experiments). **(F)** Representative images of U2OS cells expressing full-length mCherry-fusion constructs of wild-type ROCK1 or of the ROCK1mutC^PH^ mutant, stained with Alexa 488-phalloidin (F-actin) and Hoechst 33342 (DNA/nuclei). Error bars represent the standard error of the mean; *P < 0.05, ***P < 0.001, ****P < 0.0001; one-way ANOVA with Tukey’s posttest; scale bar: 10 μm. Source data are available for this figure.

To further characterize the recruitment of ROCK1 to cell contraction signal network activity, we also specifically investigated the previously identified RBD region within the coiled-coil region of ROCK1. In previous work, mutation of N1004T and L1005T was shown to strongly suppress the interaction of ROCK1 with active RhoA (Leung et al, 1996). We therefore investigated how these mutations affect the plasma membrane recruitment of ROCK1 to contraction signal network pulses, analogous to the PH domain point mutations described above. As shown in Fig S3A–E (Video 7A), the isolated RBD region itself is recruited to GEF-H1 C53R pulses, however, to a much lesser degree compared with full-length ROCK1. The N1004T and L1005T mutations completely abolished this recruitment, showing that these residues are critical for RBD-dependent recruitment to pulsatile GEF-H1/Rho activity zones (Video 7B). Introducing these mutations in full-length ROCK1 (ROCK1mut^RBD^; Fig 4F–H, middle) significantly reduced recruitment to GEF-H1 C53R pulses; however, dynamics were still clearly detectable (Fig 4F–J [middle, Video 8B]). Introducing both the mutC^PH^ and the mut^RBD^ mutations (ROCK1mut^RBD^mutC^PH^) nearly abolished ROCK1 plasma membrane recruitment (Fig 4F–J [bottom, Video 8C]). In particular, the more sensitive measurement of recruitment dynamics via the standard deviation of fluorescence intensity signals was significantly reduced for ROCK1mut^RBD^mutC^PH^ compared with ROCK1mut^RBD^. Taken together, we conclude that the canonical RBD and the PHC1 domain act synergistically to recruit ROCK1 to cortical Rho activity zones.

Video 7 Related to Fig S3. **(A)** Plasma membrane recruitment of the ROCK1 RBD region to cell contraction network activity at the plasma membrane. Time-lapse TIRF videos of mCitrine-GEF-H1 C53R and mCherry-RBD-ROCK1 in a representative U2OS cell. **(B)** Plasma membrane recruitment of the mutated ROCK1 RBD region with the N1004T/L1005T point mutations to cell contraction network activity is not detectable. Time-lapse TIRF videos of mCitrine-GEF-H1 C53R and mCherry-RBD-ROCK1-mutC^RBD^ in a representative U2OS cell. Imaging started 42 h after plating on collagen-coated glass-bottom dishes. Images were collected with a frame rate of 7.5/min. Scale bars, 10 μm. Download video

Video 8 Related to Fig 4. **(A)** Full-length ROCK1 is strongly recruited to cell contraction network activity at the plasma membrane. Time-lapse TIRF videos of mCitrine-GEF-H1 C53R and mCherry-ROCK1 in a representative U2OS cell. **(B)** Reduced plasma membrane recruitment of mutated full-length ROCK1 mutC^RBD^ with the N1004T/L1005T point mutations to cell contraction network activity at the plasma membrane. Time-lapse TIRF videos of mCitrine-GEF-H1 C53R and mCherry-ROCK1mut^RBD^ in a representative U2OS cell. **(C)** Plasma membrane recruitment of mutated full-length ROCK1 mutC^RBD^mutC^PH^ with the L1199A/F1174A/E1203K/A1292R/N1004T/L1005T point mutations to cell contraction network activity at the plasma membrane is further reduced. Time-lapse TIRF videos of mCitrine-GEF-H1 C53R and mCherry-ROCK1mut^RBD^mutC^PH^ in a representative U2OS cell. Imaging started 42 h after plating on collagen-coated glass-bottom dishes. Images were collected with a frame rate of 7.5/min. Scale bars, 10 μm. Download video

### The PHC1 domain of ROCK is critical for signal transduction to activate myosin at the plasma membrane

**Figure 5.**
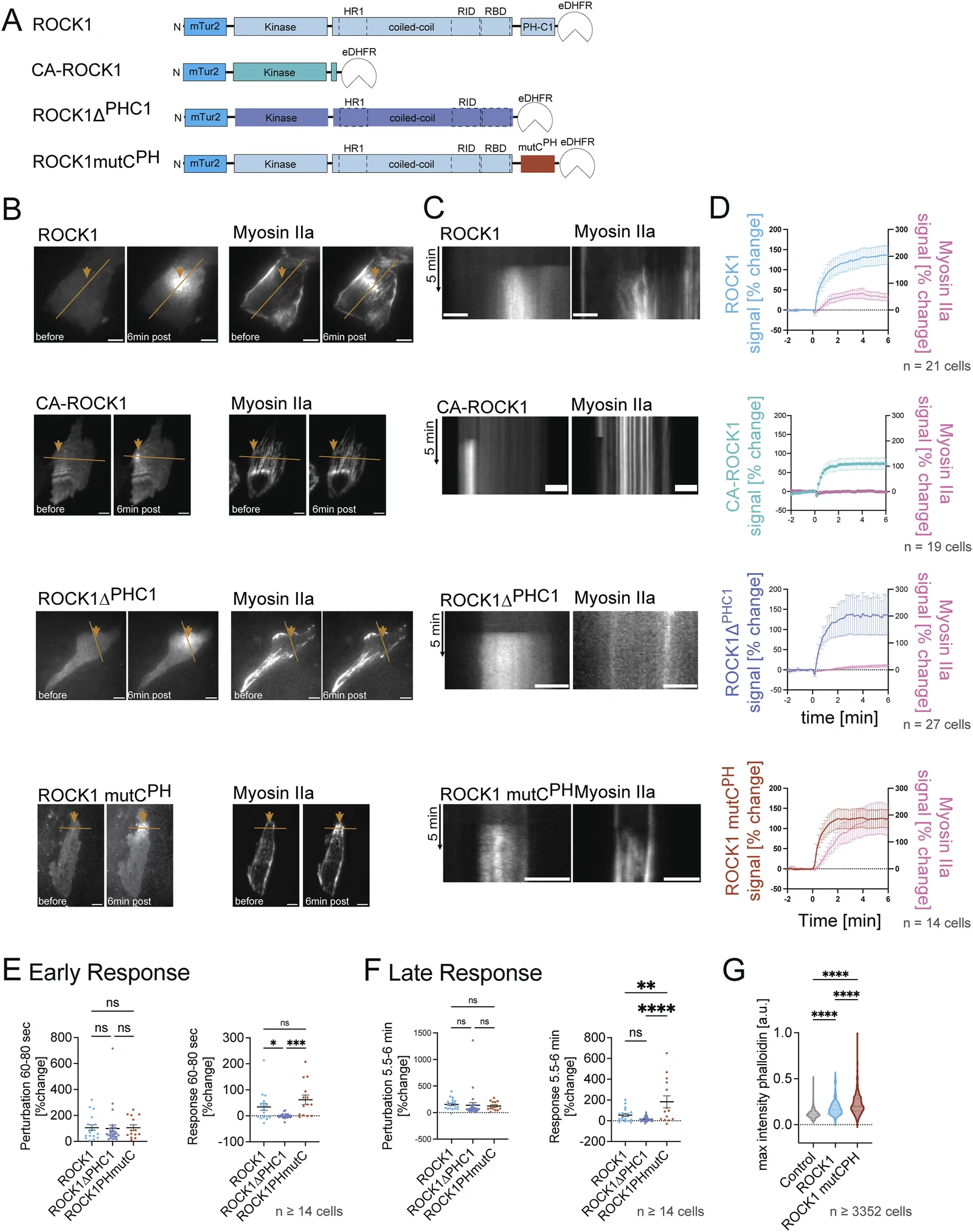
Functional role of the ROCK1 PHC1 domain in signal transduction from the plasma membrane to activate myosin II. **(A)** Schematic representation of ROCK1 constructs used in this figure (HR1: homology region 1; RID: Rho-interacting domain; RBD: Rho-binding domain; PH: pleckstrin homology; C1: phorbol ester/diacylglycerol-binding domain; mTur2: mTurquoise2; eDHFR: *E. coli* dihydrofolate reductase). **(B, C, D, E, F)** Analysis of myosin responses after local, light-induced plasma membrane recruitment of ROCK1 constructs (perturbation). **(B)** TIRF images of the mTurquoise2-ROCK1 (top, left, Video 9A), CA-ROCK1 (middle1, left, Video 9B), ROCK1Δ^PHC1^ (middle2, left, Video 9C), or ROCK1 mutC^PH^ (bottom, left, Video 9D) perturbation constructs and of the mCherry-myosin IIa response (right), before and 6 min after initiation of the light-induced perturbation. Orange arrowheads indicate the position of photodimerizer uncaging. **(C)** Kymographs corresponding to lines in (B). **(D)** Kinetics of mean fluorescence signals measured at the perturbation spot. **(E)** Mean perturbation intensity (left) and mean response (right) from 60 to 80 s post-perturbation. **(F)** Mean perturbation intensity (left) and mean response (right) from 5.5 to 6 min post-perturbation. **(G)** Maximal intensity of Atto 488-phalloidin staining of F-actin in fixed cells treated with either ROCK1 (blue), ROCK1 mutC^PH^ (red), or the empty parental construct as control (gray). Error bars represent the standard error of the mean from three independent experiments each. *P < 0.05, **P < 0.01, ***P < 0.001, ****P < 0.0001; one-way ANOVA with Tukey’s posttest; scale bars: 10 μm. Source data are available for this figure.

We next asked the question whether ROCK1-dependent myosin activation is only dependent on plasma membrane targeting, or whether additional functions of the PHC1 domain also play a role. To independently dissect these two features of ROCK1, we again employed the Molecular Activity Painting assay (Fig 3I; Chen et al, 2017). Using this method, we first recruited nonmutated, full-length ROCK1 to the plasma membrane independently of its endogenous targeting mechanism via photochemical dimerization. This artificial enrichment of ROCK1 leads to a significant increase in fluorescent-labeled myosin IIa (human MYH9, nonmuscle myosin heavy chain II-A, UniProt P35579) signals at the plasma membrane, indicating that increased local ROCK1 concentrations are sufficient to increase local ROCK1 signaling toward myosin (Fig 5A–C, ROCK1, Video 9A). Furthermore, the local myosin IIa enrichment was associated with a strong stimulation of contractile myosin flows, showing that myosin IIa is not only recruited but also activated by ROCK1 (Fig 5B, top, and Fig 5D, top, Video 9A).

Video 9 Related to Fig 5. **(A)** Rapid chemo-optogenetic plasma membrane recruitment of full-length ROCK1 leads to plasma membrane recruitment and activation of myosin IIa. TIRF video microscopy of a representative U2OS cell, expressing the perturbation construct mTurquoise2-ROCK1-eDHFR (left), the response construct mCherry-nonmuscle myosin heavy chain IIa (right), and the artificial receptor mCitrine-VSVG HaloTag-PARC. **(B)** Rapid chemo-optogenetic plasma membrane recruitment of the C-terminal truncation mutant CA-ROCK1 does not lead to plasma membrane recruitment and activation of myosin IIa. TIRF video microscopy of a representative U2OS cell, expressing the perturbation construct mTurquoise2-CA-ROCK1-eDHFR (left), the response construct mCherry-nonmuscle myosin heavy chain IIa (right), and the artificial receptor mCitrine-VSVG HaloTag-PARC. **(C)** Rapid chemo-optogenetic plasma membrane recruitment of the C-terminal truncation mutant ROCK1Δ^PHC1^ does not lead to plasma membrane recruitment and activation of myosin IIa. TIRF video microscopy of a representative U2OS cell, expressing the perturbation construct mTurquoise2-ROCK1Δ^PHC1^-eDHFR (left), the response construct mCherry-nonmuscle myosin heavy chain IIa (right), and the artificial receptor mCitrine-VSVG HaloTag-PARC. **(D)** Rapid chemo-optogenetic plasma membrane recruitment of mutated full-length ROCK1mutC^PH^ leads to increased plasma membrane recruitment and activation of myosin IIa. TIRF video microscopy of a representative U2OS cell, expressing the perturbation construct mTurquoise2-ROCK1mutC^PH^-eDHFR (left), the response construct mCherry-nonmuscle myosin heavy chain IIa (right), and the artificial receptor mCitrine-VSVG HaloTag-PARC. A focused light pulse at 405 nm was applied at timepoint 0 to target the perturbation construct to a small region of the plasma membrane. Images were collected with a frame rate of 6/min. Scale bars, 10 μm. Download video

We then tested whether the ROCK1 kinase domain by itself is sufficient to stimulate myosin activity at the plasma membrane. We performed this experiment using a perturbation construct that is based on the previously established constitutively active CA-ROCK1 mutant (Ishizaki et al, 1997), which only includes the kinase domain and a small region of the coiled coil to enable dimerization, and which was previously shown to stimulate a contractile phenotype in U2OS cells after exogenous expression (Gierse et al, 2026). Strikingly, enriching this constitutively active mutant at the plasma membrane did not lead to any significant recruitment of myosin, showing that regions of ROCK1 downstream of the kinase domain, within the coiled-coil and/or the PHC1 domains, are required for efficient myosin activation in this optogenetic assay (Fig 5B and C, CA-ROCK1, Video 9B).

Next, we specifically deleted the PHC1 tandem domain of ROCK1 to evaluate its role in myosin activation. As demonstrated in Fig 5B and C (ROCK1Δ^PHC1^), ROCK1 lacking the PHC1 domain is severely limited in its ability to activate myosin in this assay (Video 9C). Although the artificial recruitment of ROCK1 and ROCK1Δ^PHC1^ to the plasma membrane is equal, a strongly reduced corecruitment of myosin at the membrane is observed for the ROCK1Δ^PHC1^ mutant. This shows that the PHC1 domain is critical for the ability of plasma membrane–localized ROCK1 to activate myosin (Fig 5E and F).

We next investigated how the mutations that disrupt the AlphaFold-predicted PHC1-binding interface affect myosin activation by introducing the mutC^PH^ mutations into the perturbation construct. Interestingly, we found that plasma membrane targeting of ROCK1 full-length mutC^PH^ induced an even stronger myosin corecruitment compared with ROCK1 full-length wild type (Fig 5B, C, E, and F, ROCK1 mutC^PH^, Video 9D), which was again associated with strong contractile myosin flows (Fig 5 B, bottom, and Fig 5D, bottom, Video 9D). Although the artificial plasma membrane recruitment of the mutated and nonmutated forms of full-length ROCK1 was similarly efficient, we measured a more than threefold higher amount of recruited myosin IIa in the presence of these mutations. Further examination of the kinetics of myosin recruitment to ROCK1 perturbations reveals that there is no significant difference between ROCK1 and ROCK1 mutC^PH^ during the initial phase of recruitment within the first 60 s after perturbation (Fig 5D and E). In contrast, the augmented myosin activation only gets apparent at later timepoints within the following minutes (Fig 5D and F). These observations therefore suggest that the intact PHC1 domain contributes to a delayed negative regulation of ROCK1-dependent myosin activation.

We next wondered whether this inhibitory effect is dependent on the enforced plasma membrane localization of ROCK1 by the Molecular Activity Painting method and measured the response of the long-term overexpression of ROCK1 full-length constructs either in the presence or in the absence of the mutations in the PHC1 domain. Interestingly, we found that the overexpression of ROCK1 full-length mutC^PH^ stimulated the formation of strongly stained F-actin structures, which are typical for actomyosin-based contractile stress fibers. In contrast, nonmutated ROCK1 full length was much less effective in stimulating such contractile F-actin structures (Figs 5G and S3F). This observation was particularly striking considering the reduced recruitment of ROCK1 full-length mutC^PH^ to patterns of GEF-H1/Rho activity signals (Fig 4A–F).

Taken together, these experiments show that the PHC1 tandem domain is important for the signal transduction of membrane-localized ROCK1 to activate myosin (Fig 5B and C, ROCK1Δ^PHC1^). In addition, point mutations in the PHC1 domain unexpectedly enhance late myosin activation by ROCK1 at the plasma membrane, suggesting that the PHC1 region may also participate in delayed regulatory mechanisms that limit signal transduction (Fig 5E).

## Discussion

### Recruitment of the PHC1 domain to Rho activity zones

Our observations provide new insights into how ROCK1 recruitment and signal transduction are coupled to cortical Rho activity zones during myosin-dependent contraction pulses. First, we show that ROCK1 is strongly and very quickly recruited to local pulses of Rho activity at the plasma membrane with minimal temporal delay and that the isolated PHC1 domain is sufficient for this dynamic recruitment. These findings identify PHC1-dependent recruitment as an important component for the spatiotemporal regulation of ROCK1 in cells.

We believe that a direct or membrane-assisted interaction between the PHC1 domain and a membrane-associated Rho-family GTPase provides the simplest explanation that is consistent with our combined computational and cellular evidence. Three converging observations support this idea. First, PHC1 recruitment is tightly coupled to cortical Rho activity zones, suggesting that active Rho-family GTPases might contribute to this spatial recruitment. Second, AlphaFold predicts a similar putative PHC1-binding interface for several Rho-family GTPases, whereas the corresponding predictions for control complexes containing Ras-, Rap-, and Rab-family GTPases show lower confidence. Third, two independent sets of mutations targeting the predicted interface reduce PHC1 recruitment in cells, providing functional support of the predicted interaction surface. Moreover, the established electrostatic interaction of the PHC1 tandem with negatively charged membranes could cooperate with a membrane-anchored Rho GTPase to increase the local proximity of the two proteins, thereby promoting spatially selective PHC1 recruitment.

Importantly, although our investigations into the direct binding between PHC1 and active RhoA did not establish a direct interaction between the isolated PHC1 domain and RhoA, these experiments examined only one of the candidate Rho-family GTPases. Also, these experiments did not reproduce the native membrane environment, in which prenylation anchors Rho-family GTPases to the membrane. In addition, local lipids or additional cofactors may further support cooperative binding of Rho GTPases and the PHC1 domain in this native membrane environment. We therefore consider an interaction of the PHC1 domain with a membrane-associated Rho-family GTPase to be the leading hypothesis. Alternative explanations, including recruitment through another Rho-dependent cortical protein or through specialized membrane properties at Rho activity zones, remain possible, although they are not presently supported by experimental evidence.

### Potential synergistic role of the PHC1 domains and the RBDs in spatial positioning of ROCK1 in the cell cortex

**Figure 6.**
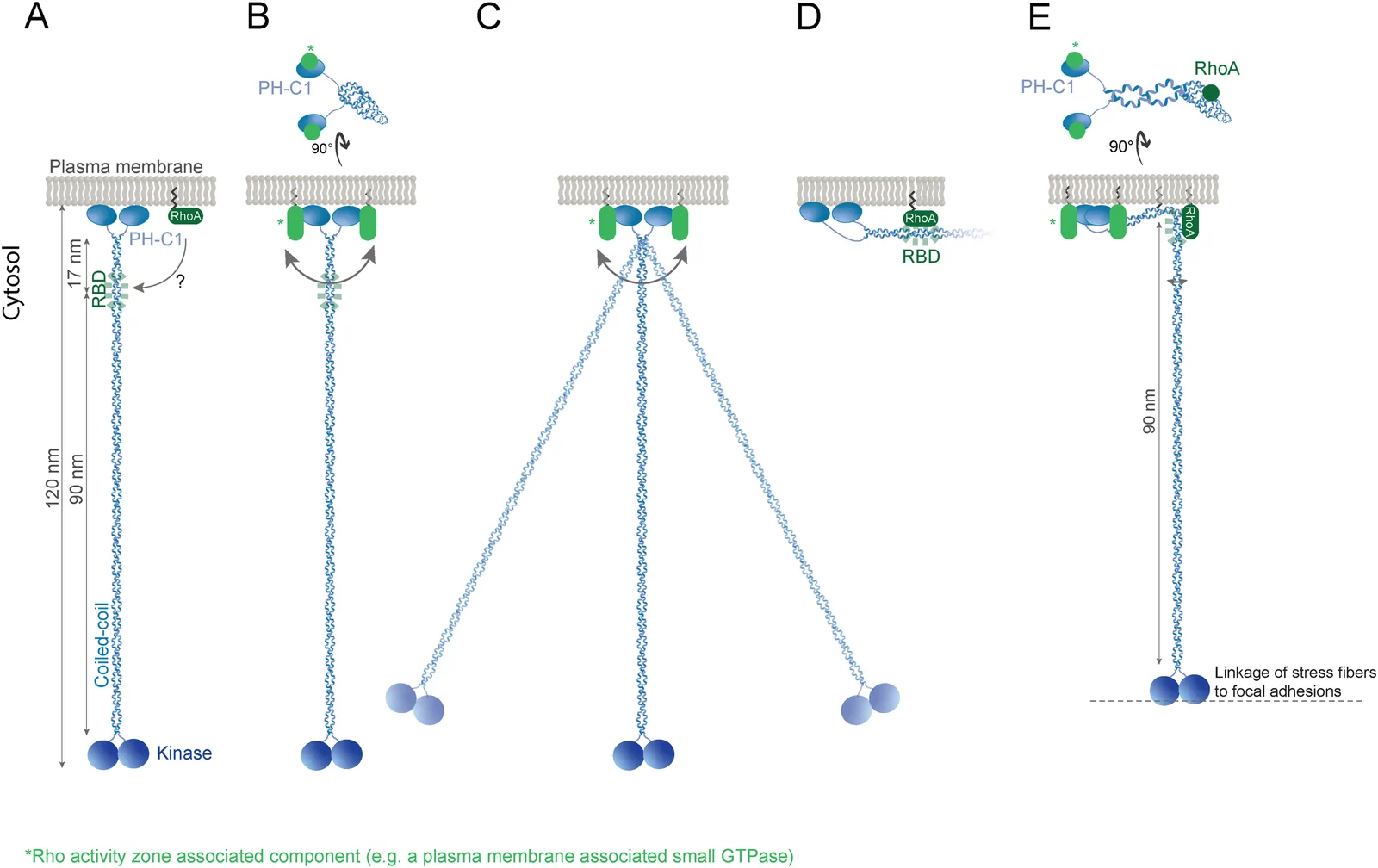
Models for PHC1-dependent ROCK1 recruitment and positioning at cortical Rho activity zones. **(A)** Schematic of “Molecular Ruler Model” (Truebestein et al, 2015). Based on this model, ROCK binds constitutively to the plasma membrane via the PH domain in its fully extended conformation. However, it is unclear how ROCK can be regulated in this model, as the previously identified ROCK1 RBD region is too far away from active Rho in the plasma membrane. **(B)** PHC1-dependent recruitment to Rho activity zones could help to reconcile this discrepancy of the “Molecular Ruler Model.” **(C)** However, because the linkers that connect the PHC1-associated membrane contacts of ROCK to the coiled-coil domain are flexible, ROCK can pivot back and forth in its extended conformation. This could effectively reduce the average distance between the ROCK1 kinase domain and the plasma membrane and thereby reduce its efficiency to activate myosin within the dense cell cortex. **(D)** Binding of ROCK1 to plasma membrane–bound Rho via its canonical RBD would stabilize an orientation of the fully extended coiled coil parallel to the plasma membrane, even further reducing the distance between the kinase domain and the plasma membrane. In this conformation, ROCK would not be able to reach myosin within the dense cell cortex. **(E)** A more stable, perpendicular orientation of ROCK1 could be realized by simultaneous engagement of the canonical RBD and PHC1-associated membrane-proximal contacts in a ROCK1 conformation that includes a predicted kink in the coiled-coil structure (see also Fig S4).

The recruitment of the PHC1 domain to Rho activity zones may help to reconcile a central discrepancy in a recent model for the regulation of ROCK: the “molecular ruler” hypothesis proposed by Truebestein et al. This hypothesis proposed that the ~90–100 nm-long coiled coil of ROCK is always in a fully elongated conformation to position the kinase domain in sufficient distance from the plasma membrane to reach myosin II motors in the dense and crowded cell cortex (Truebestein et al, 2015; Chugh & Paluch, 2018). However, in this fully elongated conformation, the plasma membrane–bound ROCK1 activator Rho is not able to reach the previously known binding site within the ROCK1 coiled coil (RBD, residues 994–1009, Fig 6A). In contrast, the PHC1 domain is positioned such that an interaction with a membrane-associated small GTPase could, in principle, contribute to ROCK1 recruitment, even if ROCK1 is present in its fully extended conformation. Thus, the PHC1 domain could help to target ROCK1 to upstream small GTPase activity within Rho activity zones to facilitate its positioning within the dense cell cortex for downstream myosin activation (Fig 6B).

However, our studies showed that the previously identified RBD region in the coiled coil also plays an important role and contributes significantly to ROCK1 recruitment to the plasma membrane. This raises the question why so many potential interaction domains for membrane-associated small GTPases exist in ROCK. Conceptually, although the PHC1 domains are sufficient to target ROCK1 to Rho activity zones at the plasma membrane, their flexible linkage to the coiled-coil domain may not by itself stabilize the proposed perpendicular orientation of ROCK1. In this configuration, ROCK may retain substantial rotational flexibility at the plasma membrane and therefore may not be efficiently positioned to reach myosin II within the dense actomyosin cortex (Fig 6C; Chugh & Paluch, 2018). The canonical RBD region in the coiled coil is even less suitable to stabilize a perpendicular orientation of the fully extended coiled coil. For example, if the RBD alone were to bind plasma membrane–associated Rho (Truebestein et al, 2015), the extended coiled-coil domain would be expected to be predominantly oriented parallel to the plasma membrane, rather than adopting the perpendicular orientation required to bridge the distance to myosin in the dense cell cortex (Fig 6D).

**Figure S4.**
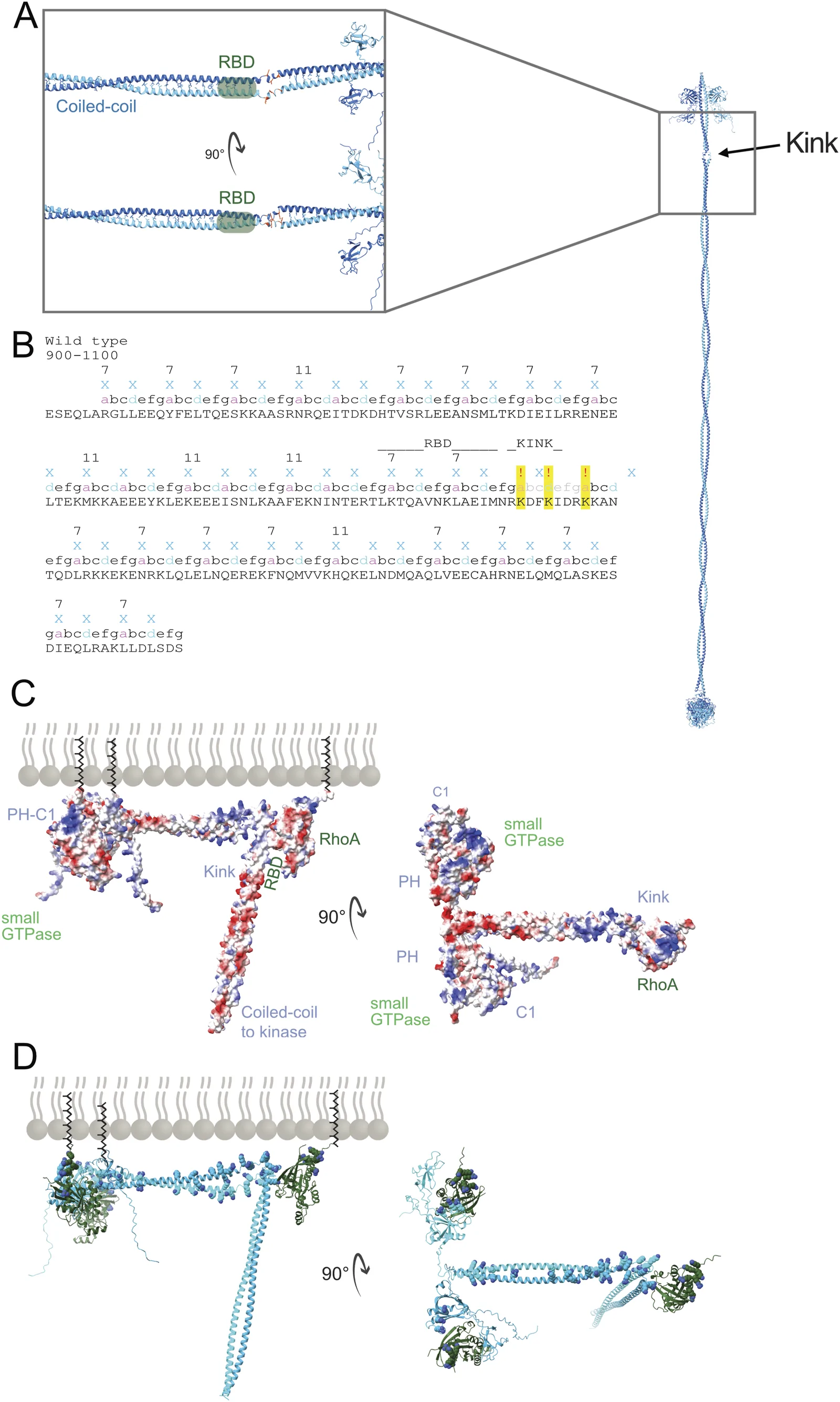
Related to Fig 6. AlphaFold prediction of a kink in the ROCK1 coiled-coil domain. **(A)** Zoom-in of a region of a full-length ROCK1 AlphaFold prediction. The full-length AlphaFold structure is based on a combination of multiple predictions of successive ROCK1 fragments (see AlphaFold methods). The predicted kink C-terminal to the RBD is due to a disruption in the repetitive coiled-coil amino sequence motif. **(B)** Amino acid sequence of the coiled-coil and predicted kink regions of ROCK1 (corresponding to AA 900–1100). “X” marks positions, in which the amino acids point toward the core of the coiled-coil structure in the AlphaFold prediction. Note that most of these amino acids have hydrophobic character. “abcdefg” represents a single, canonical coiled-coil heptad repeat, and “abcdabcdefg” represents a single, noncanonical hendecad repeat. The numbers 7 or 11 highlight the start of heptad or hendecad repeats, respectively. Positions highlighted in yellow and with an exclamation mark (‘!’) indicate amino acids that would be expected to be hydrophobic based on canonical coiled-coil heptad repeats, but instead are hydrophilic and face away from the coiled-coil core in the AlphaFold prediction. **(C, D)** Speculative structural models illustrating a possible arrangement of membrane-associated small GTPases and ROCK1. **(C)** Electrostatic surface model (positive charges are shown in blue; negative charges are shown in red). AlphaFold prediction of the RBD and PHC1 domains in a possible membrane-associated arrangement with three plasma membrane–bound small GTPases. The complex of the PHC1 domains with RhoA is shown as a representative example for its potential small GTPase interaction. A possible perpendicular orientation of ROCK1 relative to the plasma membrane is enabled by the kink and could be stabilized by multiple membrane-proximal contacts that might include the canonical RBD and PHC1 domains. The electrostatic surface of ROCK1 reveals a cluster of positively charged amino acids at the predicted membrane interaction surface. Rotating the protein by 90° reveals the full plasma membrane interaction surface and highlights the high number of positive charges (shown in blue) in this region. The predicted interaction between active Rho and the RBD region is similar to the published crystal structure (PDB ID 1S1C), which was derived from a complex of Rho with ROCK1 residues 946–1015, comprising the RBD region of the coiled coil, an upstream region of the coiled coil, but lacking most of the amino acids downstream of the RBD-binding site. **(D)** Model shown in (C) as a ribbon model with positively charged amino acids shown in dark blue.

A structural feature that may provide a mechanistic basis for the cooperative contribution of the RBD and PHC1 domain is a predicted kink in the ROCK1 coiled coil near the canonical RBD. Coiled coils are usually stabilized by repeating hydrophobic residues that face the dimer interface (McLachlan & Stewart, 1975; Stetefeld et al, 2000; Truebestein & Leonard, 2016). In the region immediately C-terminal to the RBD, this repeat pattern is interrupted, and AlphaFold predicts a kink or flexible bend in the coiled coil at this position (Fig S4A and B). In our model, this kink could allow flexibility in the angle between the upstream and downstream coiled-coil segments, potentially permitting a conformation in which at least one membrane-associated Rho molecule can bind the RBD region in the coiled coil (Figs 6E and S4C and D). Together with PHC1-mediated membrane contacts, this configuration could stabilize ROCK1 at the plasma membrane through multiple cooperative membrane-associated interactions (Figs 6E and S4C and D; compare with loose linkage in Fig 6B and C). Furthermore, clusters of positive charge at the base of the PHC1 domains (Wen et al, 2008), around the predicted kink near the RBD region, and in the helices immediately downstream of this region (AlphaFold prediction, Fig S4C and D) could provide additional membrane contacts. These contacts may further stabilize the plasma membrane association and thereby constrain the orientation of ROCK1 and favor the perpendicular configuration needed to reach myosin (Fig 6E). Based on this model, efficient activation of myosin by Rho might require the combined contribution of the PHC1 domain and the RBD to constrain ROCK1 in the perpendicular orientation.

This model is also conceptually consistent with the structural work by Hajdu et al, which proposed that Rho does not directly modulate the catalytic activity of the ROCK2 kinase domain, but instead suggested that Rho binding to the RBD induces a conformational change from a more compact to an extended state. This proposed mechanism thereby allows Rho-dependent regulation despite the large separation between the kinase domain, RBD, and C-terminal region (Hajdú et al, 2020). Additional complexity may arise from higher order ROCK1 assembly. A recent preprint by Dubey et al (2026 *Preprint*) reports structural and biochemical analyses of ROCK1, suggesting that purified ROCK1 coiled coil–containing fragments can undergo dimer–tetramer transitions. Although the relevance of these observations for cellular ROCK1 signaling remains to be established, they raise the possibility that interactions between N- and C-terminal regions could occur not only within a single ROCK1 dimer but also between neighboring ROCK1 molecules or higher order assemblies (Dubey et al, 2026 *Preprint*).

### Potential role of the PHC1 domain in transduction of upstream signals to myosin activation

Our findings raise the question whether the recruitment of ROCK1 to the plasma membrane in an extended conformation is sufficient to activate myosin II, or whether additional mechanisms might also play a role. Strikingly, we found that the artificial targeting of full-length ROCK1 via a photochemical dimerization system to a small, photoactivated region of the plasma membrane activates myosin IIa in cells, resulting in a substantial, local cell contraction response. Thus, artificial plasma membrane targeting of full-length ROCK1 is sufficient to induce a strong local downstream contractile response. This activation was dependent on several properties of ROCK1. First, artificial plasma membrane targeting of a construct that was missing most of the coiled coil did not induce detectable myosin activation, indicating that the coiled coil is essential for this response and further supporting the molecular ruler hypothesis (Fig 5B and C, CA-ROCK1). In addition, we found that the PHC1 domain also plays an important functional role, as we did not detect a substantial myosin response after artificial plasma membrane targeting of the PHC1 domain deletion mutant. Thus, both the coiled-coil and the PHC1 domain are required for efficient myosin activation after artificial plasma membrane targeting. We therefore propose that the PHC1 domain has to be intact for the correct and stable positioning of ROCK1, potentially favoring its proposed perpendicular orientation relative to the plasma membrane. Thus, plasma membrane targeting alone via an artificial domain cannot substitute all critical functions of the PHC1 domain.

An additional and unexpected role for the PHC1 domain is suggested by the observation that PHC1 mutations in full-length ROCK1 that reduce recruitment to Rho activity zones nevertheless increased the myosin response after optogenetic plasma membrane targeting. The wild-type and mutant responses were similar during the first approximately 60 s and diverged at later timepoints. This delayed divergence suggests that the intact binding interface of the PHC1 domain exerts an inhibitory influence on sustained ROCK1 signaling.

Because the mutations in the PHC1 domain were selected from an AlphaFold-predicted small GTPase interface, altered interaction with a Rho-family GTPase is one plausible explanation. Several, not mutually exclusive, mechanisms could account for the increased response.

One possibility is that the AlphaFold-predicted binding interface competes with a small GTPase that activates myosin. Based on our model, the interaction of the RBD with Rho is particularly important to stabilize the active ROCK1 orientation. In contrast, the PHC1 domain might not require a continued interaction with a small GTPase, and instead, its positive charges might be sufficient for plasma membrane tethering to stabilize the required perpendicular orientation. Potential interactions between the PHC1 domain and RhoA, RhoB, or RhoC could then compete with the RBD-Rho interaction.

Alternatively, the PHC1 domains might interact with other small GTPases that inhibit myosin activity. Apart from the classical myosin-activating signal molecules RhoA, RhoB, RhoC (Amano et al, 1996; Ishizaki et al, 1996; Kimura et al, 1996; Leung et al, 1996), and Cdc42 (Leung et al, 1998), AlphaFold also predicted potential interactions with RhoG, Rac1, RhoH, and to a lesser extent Rnd3 (Fig 3A). In particular, some of these molecules, including Rnd3 (Wennerberg et al, 2003), were previously shown to inhibit contractile signaling upstream of myosin, and could therefore contribute to the time-delayed inhibitory action of the PHC1 domain.

Although we carefully chose the alternative amino acids for AlphaFold-predicted mutations to minimize their effect on the structural stability of the PHC1 domain, we cannot exclude that they also had an effect on other potential functions of this domain. For example, the mutations could affect intramolecular interactions between the N- and C-terminal regions of ROCK that have been proposed in classical models of ROCK regulation (Fig S1).

Classical ROCK regulation models were supported by dominant-negative effects of the ROCK C-terminus in earlier biochemical and cellular studies (Leung et al, 1996; Amano et al, 1997, 1998, 1999; Ishizaki et al, 1997; Julian & Olson, 2014; Hartmann et al, 2015). However, inhibitory effects in vitro were observed most clearly with larger C-terminal fragments that included both the RBD and PHC1 regions (Amano et al, 1999), whereas smaller isolated regions were less effective or inactive in some assays (Amano et al, 1998, 1999). In addition, dominant-negative effects of C-terminal fragments varied between cellular contexts, suggesting cell type–specific modulation of the classical mechanism (Amano et al, 1997, 1998, 1999; Yasui et al, 1998). In our study, we did not detect a measurable effect of overexpressing either the isolated PHC1 or the RBD together with PHC1 on the dynamics of the cell contraction signal network (Fig 2G and H). Thus, in our experimental system, these isolated and overexpressed C-terminal fragments do not appear to exert strong dominant-negative effects on contraction-pulse dynamics. However, the absence of such an effect does not exclude intramolecular regulation of full-length ROCK1 by its C-terminal region.

Together with the observation that optogenetic targeting of full-length ROCK1 is sufficient to introduce a strong local downstream response, our findings support a model in which the C-terminal region regulates local ROCK activity primarily through membrane recruitment and conformational positioning. Furthermore, the PHC1 domain may have functions beyond membrane targeting that might involve intermolecular interactions with other proteins or intramolecular interactions with the N-terminal region and thereby might provide additional layers of regulation. Importantly, our proposed model and the alternative plausible interpretations suggest that the PHC1 domain plays an important role in regulating ROCK1 activity at the plasma membrane.

### Implications for global, cell-wide versus local, spatially restricted ROCK1 signaling

It is important to note that our observations do not imply that the PHC1 domain is generally required for ROCK1 catalytic activity. Rather, they suggest that PHC1 is specifically important for coupling membrane-positioned ROCK1 to local cortical myosin activation. This distinction is important because several previous studies showed that removal of the C-terminal regulatory region, for example, by caspase or granzyme B–mediated cleavage can generate constitutively active ROCK1 and ROCK2 (Sebbagh et al, 2001, 2005; Sapet et al, 2006). These observations demonstrate that the C-terminal region is not intrinsically required for ROCKs’ catalytic activity and that removal of this regulatory region can instead promote global ROCK activation. Similarly, the CA-ROCK1 mutant construct that was originally described by Ishizaki et al (1997) can induce global contractile phenotypes when expressed in cells, including the U2OS cell line used in this study (Gierse et al, 2026). In contrast, our optogenetic assay probes whether acute, local recruitment of ROCK1 constructs can trigger a spatially restricted myosin response at the plasma membrane. The failure of locally recruited CA-ROCK1 to induce a detectable myosin response in this assay therefore does not contradict its ability to stimulate global contractility when expressed ectopically. Rather, it suggests that local ROCK1 signaling at the plasma membrane depends not only on catalytic activity but also on the molecular geometry of ROCK1 at the cell cortex, in particular, its orientation and the access to its myosin substrate, which are mediated by additional regions of ROCK1 downstream of the kinase domain.

In conclusion, we identified the ROCK1 PHC1 domain as a recruitment and regulatory module that contributes to ROCK1 localization at cortical Rho activity zones. Our functional characterization supports an extension to the molecular ruler model, in which several regions of ROCK1, including the coiled coil, the RBD, and the PHC1 domain, cooperate to transduce signals from Rho activity zones at the plasma membrane to the distant kinase domain of ROCK1.

## Materials and Methods

### Cell culture

U2OS cells (ATCC, RRID: CVCL_0042), derived from a female donor, were cultured in DMEM + GlutaMAX (Ref.: 31966-047; Gibco) (10% FBS [Ref.: A5256701; Gibco], 10 U/ml penicillin + 100 μg/ml streptomycin [Cat. no.: P06-07100; PAN-Biotech]). HEK293T cells (ATCC RRID:CVCL_0063), a derivative of HEK293 cells of reported female fetal origin, were cultured in DMEM (P04-035550; PAN-Biotech) supplemented with 10% FCS + 100 U/ml penicillin and 100 U/ml streptomycin. Cells were incubated at 37°C in 5% CO_2_. For standard live-cell imaging, cells were plated onto 10 μg/ml collagen-I–coated LabTek glass surface slides (Thermo Fisher Scientific) 1 d before transfection. All plasmids were transfected into U2OS cells using Lipofectamine 2000 (Cat. no.: 11668019; Thermo Fisher Scientific). Sex as a biological variable was not examined experimentally, as this study used only female-derived cell lines.

### Plasmid construction

The expression construct CMV-mCitrine-GEF-H1 C53R^1^ coding for mutated GEF-H1 (*Arhgef2,* human) was generated via a Gibson assembly. Briefly, EGFP-GEF-H1 C53R (Krendel et al, 2002) was used as the backbone after restriction digestion using EcoRI and KpnI, and delCMV-mCitrine (plasmid # 214144; RRID:Addgene_214144; Addgene) (Nanda et al, 2023) was used as a template to amplify the mCitrine coding sequence using 5′-ctcgtttagtgaaccgtcagaattcgccaccatggtgagcaagggcgag-3′ as forward and 5′-ttcgatccgagacatggtacccttgtacagctcgtccatgc-3′ as reverse primers. The red fluorescent variant mApple-GEF-H1 C53R was a kind gift from Perihan Nalbant (University Duisburg-Essen). Briefly, this plasmid was generated via Gibson assembly. The mApple insert was amplified via PCR using the primers 5′-ctcgtttagtgaaccgtcagGCCACCATGGTGAGCAAG-3′ and 5′-ttcgatccgagacatggtaccCTTGTACAGCTCGTCCATG-3′, and the backbone (EGFP-GEF-H1 C53R) was digested with KpnI and EcoRI. The Rho activity sensor delCMV-mCitrine-Rhotekin-GBD was already described (Kamps et al, 2020), and the corresponding control construct delCMV-mCitrine was also constructed previously (Nanda et al, 2023). All ROCK1 and ROCK2 expression vectors and amino acid numbering are based on the canonical human isoforms (UniProt sequence Q13464 and O75116, respectively). CMV promoter–driven ROCK1 full-length expression constructs mCherry-ROCK1 and mCherry-ROCK2 were constructed via Gateway cloning using the destination vector pDEST-CMV-N-mCherry (plasmid #123215; RRID:Addgene_123215; Addgene) and the entry vector R777-E283 Hs.ROCK1 (plasmid #70567; RRID:Addgene_70567; Addgene) or R777-E285 Hs.ROCK2 (plasmid #70569; RRID:Addgene_70569; Addgene), respectively (Agrotis et al, 2019). The N-terminally truncated ROCK1 constructs were all generated by a 1-fragment Gibson assembly using mCherry-ROCK1 as a template. In all cases, 5′-ggtgccaacttttttgtac-3′ was used as the reverse primer, combined with the following forward primers: mCherry-RBD-PHC1-ROCK1: 5′-tgtacaaaaaagttggcaccatgctaaccaaagatattgaaatattaag-3′, mCherry-dPHC1 ROCK1: 5′-tgtacaaaaaagttggcaccaaaattgatagaaagaaagctaatac-3′, mCherry-mPHC1 ROCK1: 5′-tgtacaaaaaagttggcacctccggttctggaagtggaGAGTCAAGAATTGAAGGTTG-3′. Previous work already established suitable borders for split PH and C1 domains based on the ROCK1 PHC1 tandem domain (Wen et al, 2008). In the current study, the generation of the analogous split PH and C1 domains was based on these previously established sequence borders and carried out via Gibson assembly using mCherry-mPHC1-ROCK1 as the template. For mPH-ROCK1, a 1-fragment Gibson assembly was carried out with the 5′-atttccaaaatcacaaaggcccatgtaaagtaagttatgatg-3′ forward primer (containing an additional 3xGS linker sequence to connect the N- and C-terminal PH domain sequences) (Wen et al, 2008) and 5′-gcctttgtgattttggaaattag-3′ as the reverse primer. mCherry-mC1-ROCK1 was generated via a 2-fragment Gibson assembly, both fragments were obtained using mCherry-mPHC1-ROCK1 as the template. The insert was generated using 5′-cctccggttctggaagtggacatgagtttattcctacactc-3′ as forward and 5′-acaaattaagtcctctttcttatc-3′ as the reverse primer, and the backbone was amplified with 5′-agaaagaggacttaatttgttaatcaactttcttgtacaaagtggtg-3′ and 5′-tccacttccagaaccggag-3′. To create mPHC1-ROCK1 RhoA mutants in residues predicted to contribute to the predicted PHC1-RhoA interface, two double-stranded synthetic DNA fragments were synthesized (GeneStrands; Eurofins). Fragment A contained the ROCK1 coding sequence from amino acids 1119–1256 and mutations F1174A-L1199A-E1203K. Fragment B contained the ROCK1 coding sequence from amino acids 1257-1354 and the A1292R mutation. Fragment A was amplified with forward primer 5′-agtcaagaattgaaggttggctttcagtaccaaatagagg-3′ and reverse primer 5′-ctagggcagggggtggcttaaaaacatg-3′. Fragment B was amplified with forward primer 5′-taagccaccccctgccctagagtgtcgaag-3′ and reverse primer 5′-gattaactagtttttccagatgtatttttgaccactttccgg-3′. mCherry-mPHC1-ROCK1 was used as the template for the backbone. To generate mCherry-mPHC1 mutA^PH^, the backbone was amplified using 5′-tctggaaaaactagttaatcaac-3′ as forward and 5′-ctagggcagggggtggcttaaaaacatg-3′ as the reverse primer, and then, the Gibson assembly reaction was carried out with the amplified backbone and Fragment B. To generate mCherry-mPHC1 mutB^PH^, the backbone was amplified using 5′-taagccaccccctgccctagagtgtcgaag-3′ as forward and 5′-ccaaccttcaattcttgac-3′ as the reverse primer, and then, the Gibson reaction was carried out with the amplified backbone and Fragment A. For mCherry-mPHC1 mutC^PH^, the backbone was amplified using 5′-tctggaaaaactagttaatcaac-3′ and 5′-ccaaccttcaattcttgac-3′. Then, the Gibson reaction was carried out with the amplified backbone, Fragment A, and Fragment B. Full-length, CMV promoter–driven ROCK1 expression constructs that harbor point mutations in the RBDs were generated via Gibson assembly or using a QuikChange protocol. For mCherry-ROCK1 mutC^PH^, the backbone was amplified from mCherry-ROCK1 using 5′-aacatcacgaAGAGATATGCTGCTGTTAG-3′ as forward and 5′-taacgtgagcCAGTTTATCTATGTCCAATACC-3′ as the reverse primer. The insert was amplified from mCherry-mPHC1 mutC^PH^ with forward primer 5′-agataaactgGCTCACGTTAGACCTGTAAC-3′ and reverse primer 5′-gcatatctctTCGTGATGTTACATCATAACTTAC-3′. All three RBD mutant–containing plasmids, mCherry-ROCK1RBDmut^RBD^, mCherry-ROCK1mut^RBD^, and mCherry-ROCK1mut^RBD^mutC^PH^, were generated via QuikChange using 5′-cccttaaaacacaggctgttaccacattggc-3′ as forward and 5′-tcgattcattatttctgccaatgtggtaacagcct-3′ as the reverse primer, and either mCherry-ROCK1RBD, mCherry-ROCK1, or mCherry-ROCK1mutC^PH^ as templates. The “Molecular Activity Painting” perturbation construct mTurquoise2-eDHFR-GEF-H1 C53R and the dimerizer-presenting artificial receptor construct Dimerizer-PARC-CCL-mCitrine-HaloTag to locally stimulate cell contraction signal network activity were already described previously in Kamps et al (2020) and Chen et al (2002), respectively. The ROCK1 perturbation constructs mTurquoise2-ROCK1-eDHFR^2^ and mTurquoise2-CA-ROCK1-eDFHR were generated via inverse PCR with the subsequent Gibson assembly. First, the GEF-H1 C53R insert of mTurquoise2-eDHFR-GEF-H1 C53R was removed via inverse PCR using primers 5′-TAACTCAATTGTTGTTGTTAACTTGTTTATTGC-3′ and 5′-CCGCCGCTCCAGAATCTC-3′ followed by blunt-end ligation of the PCR product. The resulting plasmid was used as a backbone for the Gibson assembly after restriction digestion using XhoI (NEB). The inserts for Gibson assembly were amplified from mCherry-ROCK1 (amino acids 1–1354 for mTurquoise-ROCK1-eDHFR and 1–477 for mTurquoise2-CA-ROCK1-eDHFR) with 5′-ctggactcgtacaagatatcaatgtcgactggggacagttttg-3′ as forward and 5′-gcaatcagactgatcatagccgatccacttccagaaccggaactagtttttccagatgtatttttgacc-3′ as the reverse primer (for mTurquoise2-ROCK1-eDHFR), or 5′-gatctggactcgtacaagatatcatccggttctggaagtggatcgactggggacagttttg-3′ as forward and 5′-gccgcaatcagactgatcatagctccacttccagaaccggatagatttcttctttgatttccctcttc-3′ as the reverse primer (for mTurquoise2-CA-ROCK1-eDFHR). The mutated perturbation construct mTurquoise2-ROCK1Δ^PHC1^-eDHFR (vector ID: VB250121-1429rnx) was constructed by the company VectorBuilder starting with the mTurquoise2-eDHFR and mCherry-ROCK1 constructs described above. The mutated perturbation construct mTurquoise2-ROCK1 mutC^PH^-eDHFR was generated via Gibson assembly: The backbone was amplified from mTurquoise2-ROCK1-eDHFR with 5′-aacatcacgaAGAGATATGCTGCTGTTAG-3′ as forward and 5′-taacgtgagcCAGTTTATCTATGTCCAATACC-3′ as the reverse primer, and the insert was amplified from mCherry-mPHC1 mutC^PH^ with forward primer 5′-agataaactgGCTCACGTTAGACCTGTAAC-3′ and reverse primer 5′-gcatatctctTCGTGATGTTACATCATAACTTAC-3′. The expression vector mCherry-NMHC2a encoding nonmuscle myosin heavy chain IIa (*MYH9*, human) was already described previously (Kowalczyk et al, 2022).

### Microscopy

TIRF microscopy was performed on an Olympus IX-81 microscope, equipped with an Apo TIRF 60 × 1.45 NA oil immersion objective, a TIR-MITICO–motorized TIRF illumination combiner, and a ZDC autofocus device. Four different lasers were employed for imaging cells: a 514-nm OBIS diode laser (150 mW) (Coherent, Inc.) and Cell R diode lasers (Olympus) with wavelengths of 405 nm (50 mW), 445 nm (50 mW), and 561 nm (100 mW). In addition, wide-field illumination was performed using the Spectra X light engine (Lumencor). Signals were detected using an EMCCD camera (C9100-13; Hamamatsu) at medium gain without binning. Live-cell imaging was carried out at 33°C in CO_2_-independent Hepes-stabilized imaging medium (Cat. no.: P04-01163; PAN-Biotech) supplemented with 10% FBS and at the indicated frame rates. For imaging of F-actin, U2OS cells were fixed with 4% PFA in PBS for 15 min, followed by permeabilization using 0.25% Triton X-100 in PBS for 10 min, blocking with 2% BSA in PBS for 1 h at room temperature, and staining with phalloidin-Atto 488 (1:1,000 dilution, 49409; Sigma-Aldrich) and counterstaining of nuclei with Hoechst 33342 (1:1,000 dilution, Sigma-Aldrich) for 1 h at room temperature.

### Functionalized Molecular Activity Painting

The original Molecular Activity Painting method was described previously (Chen et al, 2017), and a simplified variant used in this work was developed subsequently (Kamps et al, 2020). Briefly, 1.0–1.4 × 10^5^ U2OS cells were plated into each well of a 6-well plate (Cell+, Ref.: 83.3920.300; Sarstedt) and transfected on day 2 with a dimerizer-presenting artificial receptor construct (dimerizer-PARC), a construct containing eDHFR fused to a protein of interest, and fluorescent-labeled readout protein(s). On the next day, the cells were detached by incubation with 10 mM EDTA (in DPBS, pH 7.4) for 25–30 min without trypsin or by incubation with the Enzyme Free Cell Dissociation solution (S-014-B; EMD Millipore Corp) for 10 min. The cells were washed 2× (EDTA detachment) or 1x (Enzyme Free Dissociation Solution detachment) with serum-free medium followed by centrifugation. After washing, cells were resuspended in 200 μl serum-free medium and plated onto the functionalized glass-bottom dishes (see next paragraph). After incubation at 37°C, 5% CO_2_ for 30 min, 200 μl 20% FBS-containing medium was added and incubation continued overnight.

On day 2, glass-bottom dishes (8-well LabTek I, VWR) are coated with 0.1% poly-L-lysine hydrobromide (Sigma-Aldrich) at 4°C overnight. The next day, the wells were washed 3× with DPBS followed by biotinylation of adsorbed poly-L-lysine using 1 mg/ml EZ-link Sulfo-NHS-Biotin (Thermo Fisher Scientific) in DPBS for 1 h at room temperature. During incubation, two solutions were prepared on ice (the required volume was calculated for three wells): (i) streptavidin solution: 2 μl streptavidin ( 1 mg/ml; Serva) + 1 μl streptavidin Alexa Fluor 750 (Life Technologies, 0.1 mg/ml) + 12 μl DPBS and (ii) antibody solution: 3 μl biotin-labeled anti-VSVG antibody (ab34774; Abcam; RRID: AB_778903, 1 mg/ml) + 12 μl DPBS. After three washes with DPBS, all remaining DPBS was aspirated from the biotinylated glass surface. The two prepared solutions were quickly mixed, and 5 × 2 μl drops were added on the dry glass surface of each well to finalize functionalization by streptavidin and antibody addition in the resulting small circular patterns (diameter: 1–2 mm). After a minimum incubation period of 2 min, the wells were washed 3× with DPBS and then stored at 4°C, and submerged in DPBS, for up to 45 min before cell seeding. The fluorescence signal of Alexa Fluor 750 enabled the identification of functionalized regions during imaging.

Covalent labeling for chemo-optogenetic perturbation was carried out by incubating 20 μM of the Nvoc-TMP-Cl photocaged chemical dimerizer (Chen et al, 2017) in Hepes-stabilized imaging medium for 60 min. Excess dimerizer was removed by three consecutive washes with Hepes-stabilized imaging medium, followed by a fourth wash after an additional 30-min incubation. Photo-uncaging was achieved using a 405-nm laser focused to a spot through the FRAP mode of the TIR-MITICO–motorized TIRF illumination combiner. The process was carried out for 400 ms at 180 nW (20% laser power), with the laser power measured at the 60× objective. Because the perturbation is irreversible, only a single light pulse was applied to each cell.

### Image and video analysis

All image analysis was conducted using ImageJ (https://imagej.net/ij/; RRID:SCR_003070), and figures were created with Adobe Illustrator (RRID:SCR_010279). Kymograph analysis was performed using the multi kymograph plugin in ImageJ. Kymographs and the corresponding line regions were only used for data visualization and were not used for any of the statistical analyses. Cell movies were stabilized using the ImageJ plugins Image Stabilizer and Image Stabilizer Log Applier (https://imagej.net/plugins/image-stabilizer). Statistical analyses and generation of plots were carried out with Prism (GraphPad; RRID:SCR_002798). All statistical tests were two-sided.

### Analysis of perturbation response kinetics

The kinetics of Rho, GEF-H1, ROCK1, or myosin fluorescence signal changes after chemo-optogenetic perturbations were measured at the local perturbation site, as described previously (Chen et al, 2017). The fluorescence signal profiles for the perturbation and response, shown in Figs 3 and 5, are normalized to the timepoint right before perturbation. The corresponding mean perturbation and response levels were averages of the 3 timepoints in the time ranges of 10–30 s (Fig 3M), 60–80 s (Fig 5E and F, early response), or 5.66–6 min (Fig 5E and F, late response).

### Analysis of cell contraction signal network dynamics

As an unbiased analysis of signal network dynamics, we employed a custom-made ImageJ macro as previously described (Graessl et al, 2017) to determine the standard deviation, pulse frequency, and pulse amplitude of proteins that are dynamically recruited to the plasma membrane with minor modifications.

### AlphaFold modeling

**Figure S5.**
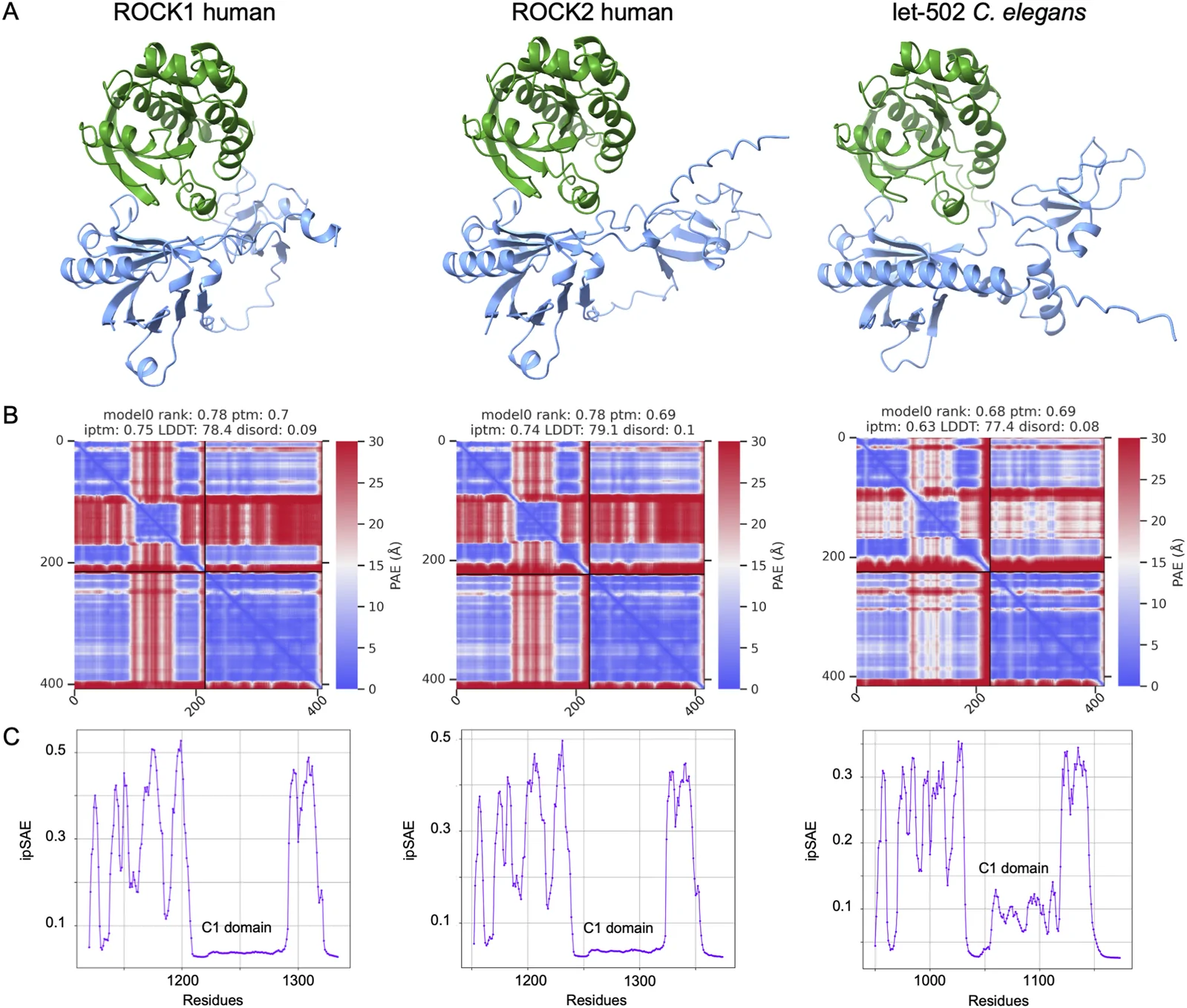
Related to Materials and Methods. AlphaFold predictions of the complex of RhoA with ROCK PH domains. **(A)** Predicted structures of the ROCK PH domain (blue) in complex with human or *C. elegans* RhoA (green), using AlphaFold (Abramson et al, 2024). **(B)** PAE plots for the complexes shown in (A). **(C)** ipSAE plots of the predictions shown in (A).

The model of human ROCK1 kinase (UniProt Q13464) was created by predicting dimers of the fragments 1–600, 500–700, 600–800, 500–1,000, and 900–1,354 using AlphaFold Multimer version 2.2.0 (Evans et al, 2021 *Preprint*) and joining them together using coot (RRID:SCR_014222) (Emsley & Cowtan, 2004), followed by geometry relaxation with alpha-helical constraints. For models of possible complexes between RhoA and the ROCK PHC1 domain, fragments 1120–1334 of ROCK1, 1152–1374 of ROCK2 (UniProt O75116), and 950–1173 of let-502 from *C. elegans* (UniProt P92199) were predicted using AlphaFold3 (Abramson et al, 2024) in complex with human or *C. elegans* RhoA (UniProt P61586 or Q22038) (Fig S5A). The PAE and LDDT scores were very significant for all three complexes. The PAE plot reveals a stronger connection between the C1 domain and RhoA for the let-502 compared with the human PHC1 domains, suggesting that in the model, the main predicted interface for the human proteins is the PH domain, although some residues of the C1 domain might also contribute (Fig S5B). The predicted RhoA-PHC1 interface in the *C. elegans* proteins has therefore a slightly different residue conservation pattern. This is also visible in the ipSAE plots (Dunbrack, 2025 *Preprint*), where the best *C. elegans* model shows again higher scores for the C1 domain (Fig S5C). Corroborating this observation, the different models for the *C. elegans* protein predict the C1 domain in quite similar positions, whereas the relative orientation of the C1 domain compared with the PH and RhoA domains varies. Still, there are some residues with good PAE scores relative to RhoA, and the steric positioning of RhoA by the C1 domain might also contribute to the overall affinity. For the construct mPH-ROCK1 where the two sections of the PH domain are connected by a long linker, AlphaFold3 predicts the same complex as for the construct that includes the ROCK1-C1 domain, but with lower ipTM scores (0.75 versus 0.25), indicating that the C1 domain indeed contributes to the stability of the predicted complex. For predictions comparing various small GTPases (Fig 3A), the human full-length GTPase sequences and the human ROCK PHC1 domain (AA 1101–1354) were used to calculate ipTM values using AlphaFold3 (Abramson et al, 2024).

The putative membrane-bound model (Fig S4C and D) was built by superimposing an AlphaFold2 prediction of human ROCK1 residues 900–1354, the crystal structure of human ROCK1 residues 946–1015 (PDB ID 1S1C), and predictions of ROCK1 900–1354 in complex with RhoA. The predicted PHC1-RhoA complex was oriented manually so that the RhoA C termini and the positive charges of the PHC1 domains face the putative plasma membrane surface, which is easily achieved because of the disordered linker formed by residues 1103–1121 between the coiled coil and the start of the PH domain of ROCK1.

### Pull-down assay in HEK293T cells

1 d before transfection with expression vectors for pull-down experiments, HEK293T cells were seeded at 30–35% density on 10-cm cell culture dishes (Greiner CELLSTAR dish 100 × 20 mm, Ref: 664160). On the next day, cells were transfected with 5 μg of the different mCherry and GFP constructs, except for mCherry-ROCK1 full length of which 7.5 μg was transfected. 300 μl Ham’s F10 (L0140-500; Biowest) was added to the DNA, and 3 μl PEI/1 μg DNA were diluted in 300 μl F10. Next, the PEI- and DNA-containing F10 solutions were combined and vortexed. After incubation for 20 min, the transfection mixtures were added to the cells. On the next day, the GFP affinity purification was performed according to the ChromoTek GFP-Trap Magnetic Particles M-270 Co-IP protocol 24 h post-transfection.

After pulldown, the collected aliquots of input and pull-down samples were subjected to SDS–PAGE. PageRuler Plus Prestained Protein Ladder (10–250 kD) was used as a molecular weight marker. Next, proteins were transferred onto a nitrocellulose membrane (Amersham Protran Premium 0.45 μm NC, 10600003) in 1× transfer buffer (10×: 1 liter H_2_O + 144 g glycine + 30.4 g Tris) + 20% methanol at 4°C and 20–30 V for 16 h. After transfer, blots were blocked using blocking buffer (1x PBS + 0.05% Tween-20 + 2% BSA) for 1 h. Next, blots were incubated with primary antibodies that recognize mCherry (ab125096; Abcam; RRID:AB_11133266) or GFP (ab290; Abcam; RRID:AB_303395) at a dilution of 1:2,000 in blocking buffer for 1 h. After washing 3× with washing buffer (1x PBS + 0.05% Tween-20) for 5 min, incubation with secondary antibodies (IRDye 680RD Goat anti-Mouse, 926–68070; RRID AB_10956588 and IRDye 800CW Goat anti-Rabbit, 926–32211; RRID AB_621843) diluted 1:10,000 in blocking buffer was performed for 1 h. Subsequently, blots were washed 3× with washing buffer and imaged using a LI-COR Odyssey imager.

### Additional information relevant for reproducibility

Randomization was not used to assign cells to experimental groups, because the study did not involve animal cohorts or treatment groups requiring formal random allocation. For microscopy-based experiments, fields of view were selected without prior knowledge of the quantitative outcome, based on the presence of transfected, morphologically healthy cells suitable for imaging. Where applicable, all analyzable cells in the selected fields of view were included in the analysis, unless technical criteria such as failed transfection, loss of focus, severe cell damage, or insufficient image quality precluded reliable quantification.

All analyses were based on predefined image-processing and quantification procedures applied consistently across conditions, including unbiased analysis of fluorescence dynamics using ImageJ-based workflows. Blinding was not considered necessary, as quantitative readouts were derived from the recorded image data using the same criteria for all experimental conditions.

No formal statistical power analysis was performed to predetermine sample size. Instead, sample sizes were chosen based on experimental feasibility, previous experience with comparable live-cell imaging and perturbation experiments, and the aim to include sufficient numbers of analyzable cells or independent experiments for robust estimation of the measured effects. The number of cells or independent experiments that were analyzed is reported in the corresponding figure legends.

Cell line authentication was not performed by STR profiling for this study. U2OS and HEK293T cells were maintained under standard culture conditions and routinely monitored for expected morphology, growth behavior, and transfection performance during experiments. Only cultures showing normal morphology and experimental behavior were used.

No substantial new analysis software was developed for this study. All image analysis was performed using ImageJ and previously established ImageJ plugins or macros, as described above, and statistical analysis and plotting were carried out with GraphPad Prism. AlphaFold-based modeling was performed using the indicated published AlphaFold versions and associated structural analysis tools. No additional custom code requiring public deposition was generated for this study.

Detailed experimental procedures are provided in the Materials and Methods section. No separate online protocol was generated for this study.

## Supplementary Material

Reviewer comments

## Data Availability

Source data are included. All other data are available from the corresponding author upon reasonable request.

## Footnotes

^1^
This construct was primarily developed and generated in a companion study by Gierse et al (2026) (https://doi.org/10.1091/mbc.E26-01-0026). The experimental details for the construction of this construct are briefly stated in both studies. For future derivative work that uses CMV-mCitrine-GEF-H1 C53R, the companion study should be cited.

^2^
This construct was primarily developed and generated in a companion study by Gierse et al (2026) (https://doi.org/10.1091/mbc.E26-01-0026). The experimental details for the construction of this construct are briefly stated in both studies. For future derivative work that uses mTurquoise2-ROCK1-eDHFR, the companion study should be cited.
