# Supplementary material for: The ROCK1 PHC1 domain mediates recruitment to Rho activity zones and contractility signaling
Source: Life Sci Alliance. 2026 Oct 6;9(12):e202603885. doi: 10.26508/lsa.202603885 (PMC13646499; doi:10.26508/lsa.202603885)
Supplement: Supplementary file 1 [file LSA-2026-03885_SdataFS2.1.pdf]

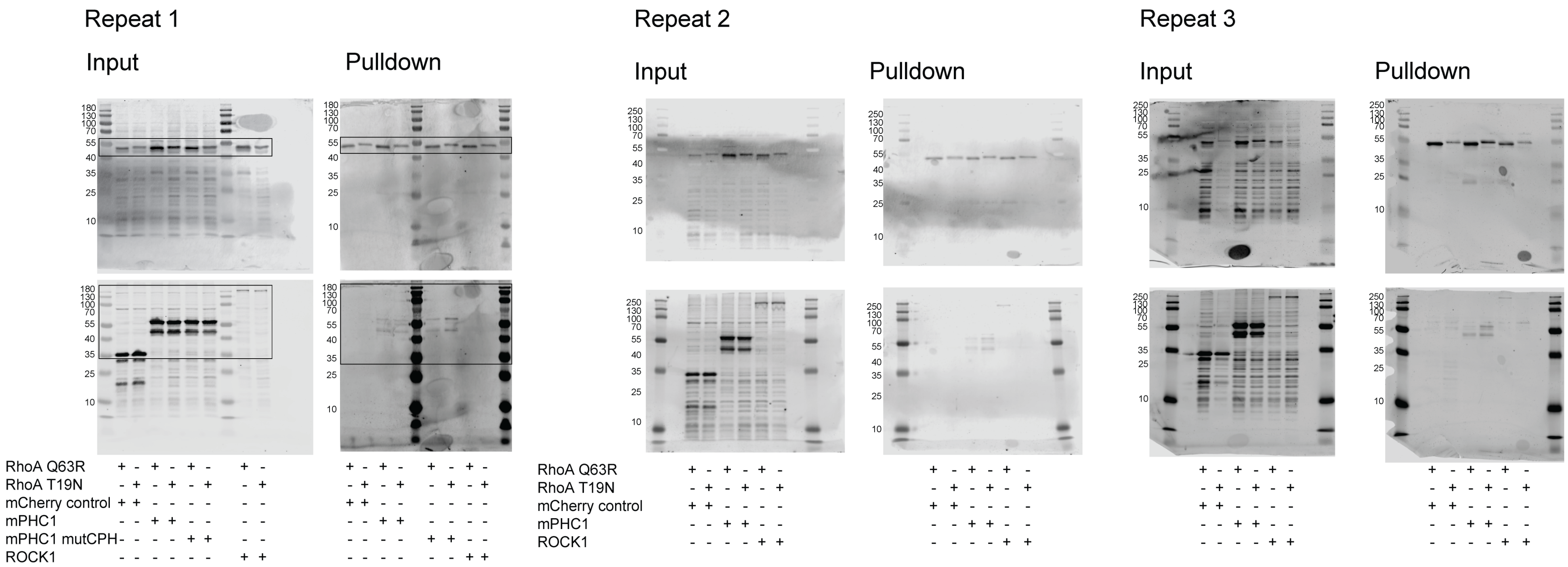

Repeat 1
Input
Pulldown
Repeat 2
Input
Pulldown
Repeat 3
Input
Pulldown
RhoA Q63R
RhoA T19N
mCherry control
mPHC1
mPHC1 mutCPH
ROCK1
RhoA Q63R
RhoA T19N
mCherry control
mPHC1
ROCK1
