# Supplementary material for: The ROCK1 PHC1 domain mediates recruitment to Rho activity zones and contractility signaling
Source: Life Sci Alliance. 2026 Oct 6;9(12):e202603885. doi: 10.26508/lsa.202603885 (PMC13646499; doi:10.26508/lsa.202603885)
Supplement: Supplementary file 2 [file LSA-2026-03885_review_history.pdf]

The ROCK1 PHC1 domain mediates recruitment to Rho activity zones and contractility signaling

Carolin Gierse, Ingrid Vetter, Donna Schweizer, and Leif Dehmelt
DOI: https://doi.org/10.26508/lsa.202603885

Corresponding author(s): Leif Dehmelt, TU Dortmund University

Review Timeline:

Submission Date:	2026-08-19
Editorial Decision:	2026-08-25
Revision Received:	2026-09-14
Accepted:	2026-09-16

Scientific Editor: Sarita Hebbar

Transaction Report:

(Note: With the exception of the correction of typographical or spelling errors that could be a source of ambiguity, letters and reports are not edited. The original formatting of letters and referee reports may not be reflected in this compilation.)

Please note that the manuscript was previously reviewed at another journal and the reports were taken into account in the decision-making process at *Life Science Alliance*.

Reviews

Reviewer #1 Review

Comments to the Authors (Required):

The manuscript by Gierse and colleagues employs an experimental model previously developed in their laboratory, which relies on overexpression of a microtubule-binding-deficient mutant of the Rho-GEF GEF-H1. This system generates cortical contraction pulses at the cell membrane. These pulses recruit active RhoA and ROCK1 to the plasma membrane through a signaling pathway previously described by the authors. This pathway combines a rapid positive feedback loop, mediated by the interaction between GEF-H1 and RhoA, with a slower negative feedback loop mediated by Myosin II.

In this resubmission, the authors propose that the recruitment of ROCK1 to RhoA active zones depends both on the canonical RBD domain and the C-terminal PHC1. The C-terminal PHC1 has not been previously characterized to respond to RhoA activation and the authors show that it can be recruited to RhoA active zones independently of the RBD. In the first submission the authors proposed that PHC1 was a new RhoA binding site in ROCK1, supported by AlphaFold2 modeling. However, this was not proven, either in vitro or in vivo. In the resubmission the authors tried unsuccessfully to show that RhoA binds to PHC1. The manuscript still shows that recruitment of PHC1 is RhoA dependent, but the mechanism is unclear and based on the current evidence rules out a direct interaction with RhoA. As mentioned in my initial review, this has an impact on the significance of the findings.

Major points:

-In the absence of proof of direct interaction with RhoA, some of the original text and figures have now a different meaning or require a new interpretation. The authors still propose in Fig 5 and in their model (6E and F) a direct interaction with RhoA. In addition, AlphaFold is used to predict the residues that interact with RhoA, which are then used to generate mutations. How are the effects of these mutations interpreted now? What is the role of these residues if they are not interacting with RhoA?

-The authors have toned down parts of the text and the discussion related to RhoA interaction, but based on the model (Fig 6) and Fig 5 they seem to be still proposing this is a direct interaction which they cannot detect it because of technical reasons.

-In the Discussion (p15) the authors mention that "PHC1 domain mediates context dependent recruitment to cortical RhoA activity zones". It is not clear what "context dependent" means, and there is no data provided to support that.

-It is not clear why the deltaPHC1 domain and the mutPHC1 domain constructs have such different responses (no Myosin activation in the deletion mutant and increased activation in the mutant). Both should be deficient in targeting. The authors propose that PHC1 may function in a negative feedback loop possibly by regulation ROCK1 targeting or positioning relative to RhoA or Myosin II. If this is the case, both mutants should be impaired in targeting in a similar way.

-There are no significant changes in the resubmission compared to the first version, other than toning down the role of a direct interaction with RhoA.

Minor:

-There is an over emphasis on the manuscript that PHC1 is "sufficient" to recruit ROCK1 to the membrane. I think this is misleading. As a single domain this is correct. However, the data shows that RBD is still the major driver for ROCK1 membrane recruitment and ROCK1 is recruited much less efficiently when RBD is mutated, suggesting PHC1 is a minor contributor.

-In p14, I believe 5D should be 5E, 5E>5F and 5F>5G

-In Fig 5 it would be informative to see representative images of the changes in actin staining and not only the quantification (Fig 5G)

Reviewer #2 Review

Comments to the Authors (Required):

The new revision provided by the authors constitutes some improvement over the original version, chiefly by 1) clarifying that the direct interaction between the PHC1 domain and RhoA-GTP could not be shown biochemically and, thus, remains mechanistically unresolved, and 2) providing discussion of additional relevant literature, mainly Hajdu et al. and the new preprint from the Ahmadian group, Dubey et al.

Together with the above two sources, the manuscript by Gierse et al. paints a convoluted and, at times, confusing picture. Thus, the controversy about the in vivo position of ROCK1 on the membrane remains open. Some results of Gierse et al. and the new results by Dubey et al. seem to support the "perpendicular" hypothesis of Trubestein et al., 2015, while others are consistent with the proposal by Hajdu et al.

My first question to the authors was to clarify their position on the role of active RhoA in activation of the kinase activity of ROCK1. To this the authors replied that "We did not intend to convey that Rho is not essential for ROCK1 activation, but based on this data instead propose that recruitment of ROCK1 to the plasma membrane is sufficient for ROCK1 activation." This message, once distilled, precisely means that RhoA is NOT necessary for the activation but for localization and the fact remains that FL ROCK1 recruited to the membrane without any participation of RhoA (optogenetically) is fully catalytically active. However, data on the recruitment of FL ROCK1 minus the PHC1 domain suggests that PHC1 plays inhibitory role. These results are still puzzling and the authors did not provide any new experimental clarifications or plausible hypotheses explaining the conundrum.

In summary, the main messages of this paper are 1) the PHC1 domain plays an important role in localization of ROCK1 to the RhoA activity zone and, potentially, some role in the activation of ROCK1 kinase activity; 2) the classical autoinhibition mechanism of ROCK kinases via interaction between the N and C termini is put in question. Other observations by the authors are mostly confirmatory and have been already published by the authors and others.

Reviewer #1 Review

Comments to the Authors (Required):

The manuscript by Gierse and colleagues employs an experimental model previously developed in their laboratory, which relies on overexpression of a microtubule-binding-deficient mutant of the Rho-GEF GEF-H1. This system generates cortical contraction pulses at the cell membrane. These pulses recruit active RhoA and ROCK1 to the plasma membrane through a signaling pathway previously described by the authors. This pathway combines a rapid positive feedback loop, mediated by the interaction between GEF-H1 and RhoA, with a slower negative feedback loop mediated by Myosin II.

In the present study, the authors propose that a new RhoA-binding site in ROCK1 facilitates its recruitment to the plasma membrane upon RhoA activation by GEF-H1. This site, located in the C-terminal PH domain, has not been previously identified. The authors further suggest that this newly described interaction contributes to downstream myosin activation by acting synergistically with the canonical RBD domain, positioning ROCK1 at the plasma membrane in a manner that promotes Myosin II activation.

The model system utilized allows for precise perturbations of the system, which the authors utilize to identify the domains in ROCK1 that mediate the recruitment. In addition, the use of an optogenetic system allows them to control the recruitment of ROCK1 constructs to the membrane in space and time.

Overall, the experiments are sound and thorough and systematically dissect the pathway. The results are novel and provocative, and may shed light on some previously contradictory or ambiguous findings. However, there are also important concerns.

The main issue in this manuscript is that, even though this new RhoA binding site is proposed, is never directly validated. Recruitment of ROCK1 to the membrane mediated by this domain (PHC1), correlates with GEF-H1 mediated activation of RhoA, but the authors provide no evidence that this occurs through a direct interaction with RhoA. There is also no evidence that the interaction with Rho is nucleotide dependent. Without confirmatory biochemical or biophysical experiments, the author's conclusions, even though supported by the data, remain speculative.

There are several alternative explanations for the observed recruitment of ROCK1 via PHC1. The recruitment could still be Rho-GTP-dependent, as the data suggest, but not necessarily mediated through direct RhoA binding. For example, PHC1 could interact with a Rho effector, with specific membrane lipids, or with GEF-H1 itself. Because this proposed interaction is central to the manuscript's conclusions, direct validation is essential. Without it, the authors would need to rewrite the manuscript, toning down the interpretation and significance of their findings to reflect this uncertainty. The absence of this evidence also decreases the significance of their findings.

In summary, this is an interesting and well-executed study that addresses an important and timely question in RhoA-ROCK signaling dynamics. However, the lack of direct biochemical evidence for the proposed RhoA-PHC1 interaction represents a major weakness. Addressing this point is essential, in my opinion.

Other concerns/comments.

-In most experiments, a line is drawn across the cell to generate kymographs, and a small Roi is used to measure the intensity changes of the different markers over time. Judging by the images provided, where these lines/ROI may affect the outcome of the measurements. How were these positions chosen for each experiment?

- The manuscript would benefit from additional information on the number of biological replicates and cells analyzed in each experiment. Ideally, individual experimental means should be shown (e.g., in Fig. 2E-H).

-Several movies are included in the supplemental material, but these are not referenced in the main text or figure legends. The reader has no way of knowing they exist.

-Details on statistical analyses are lacking. While some figures display p-values, there is no information on the type of test used in each case.

- In Figure 5B (CA ROCK1), the example cell shown is of very poor quality (the accompanying movie as well). The signal from the expressed constructs appears much weaker than in other conditions (presumably this is among the better cells imaged). This raises concerns about differences in expression levels among constructs and how these might affect the results. There is no indication that the authors controlled for expression levels-by quantifying intensity, generating stable lines, or using Western blotting. This is a limitation, especially considering that some experiments analyze relatively few cells (e.g., as few as 14).

Minor issues

-In p2, second paragraph (Introduction), I believe "Figure 4A" should be replaced by "Figure 1A"

-In p11 last line, "Figure C-D" is missing the figure number

-In p12 last paragraph, second line, I believe "mutAPH is the single mutant" and "mutBPH" is the triple mutant, not the other way round as stated.

-in Figure 5E-F is not clearly what mean perturbation intensity and mean response mean and how it was measured. One can infer that one is the recruitment of each of the ROCK1 construct and the other was myosin recruitment, but details are insufficient.

Reviewer #2 Review

Comments to the Authors (Required):

The manuscript by Gierse et al. provides interesting novel results and analysis, which both shed a new light on the enigmatic nature of ROCK1 biology and raise new questions that need to be addressed.

The authors start with the demonstration that ROCK1 and 2 are recruited to the pulses of actomyosin contraction induced by CA GEF-H1. They then proceed to show that all ROCK fragments previously described as ROCK domains interacting with active Rho are dispensable for the recruitment of ROCK1 into the pulses of contraction. The authors demonstrate that the C-terminal PH domain split by C1 domain (PHC1 in their nomenclature), in fact, interacts with active Rho providing two additional C-terminal membrane-binding sites to the ROCK1 kinase dimer. They further identify specific residues responsible for the binding of PHC1 and Rho and generate a mutant (mutC PH) that fails to interact with Rho. Interestingly, the authors find that the "classical" RBD proposed to interact with active Rho in the earlier literature, still plays that role and can be engaged by the membrane-bound Rho at the same time as the C-terminal PHC1 domains, despite being located 17nm away from the ROCK C-terminus along the dimeric CC domain. Satisfyingly, only a mutant in both PHC1 and RBD domains fails to be recruited to the contraction pulses.

These novel results are important and lay to rest some earlier controversies (Trubestein et al, 2015). An additional related result (see more below) is mentioned somewhat in passing, only in the Discussion section (should be Conclusion as the authors have main results labeled as Results and Discussion).

Major issue.

While the above results pertaining to the role of ROCK1 interaction with Rho for localization to the membrane appear to be clear and logical, the proposed by the authors role of Rho in the activation of ROCK1 opens new major controversies in the field.

In this section, the authors use their in-house-built optogenetics system to recruit various ROCK1 constructs. Optogenetic recruitment of FL ROCK1 resulting in myosin-2 activation suggests that active Rho is not essential for ROCK1 activation, although Fig. 5D might hint that the activity of the recruited kinase is not optimal.

Then the authors show that ROCK1-delta PHC1 has no activity once recruited in the same fashion. Moreover, FL ROCK1 mutated in PHC1 binding to Rho (mutC PH) is 3 times more active than WT ROCK1. These results are highly surprising in the light of earlier published results that show that the interaction of PHC1 domain and the N-term of ROCK1 is autoinhibitory and is relieved by binding to active Rho. The authors mention one original paper in conjunction with this result (Leung et al., 1996) and cite an obscure chapter (Khalil 2010), which this reviewer could not find on the internet. The authors are sent to more popular and easily accessible reviews (Julian and Olson, 2014 Pubmed 25010901; Hartmann et al., 2015 /26635606/) that introduce the primary literature supporting ROCK activation by Rho. Those results clash with the newly presented by the authors results on OG recruitment. For example, the results by Gierse et al. contradict a large body of evidence showing that, in apoptosis, caspases produce constitutively active ROCK1 and 2 by precisely removing the C-terminal PHC1 domain. In authors picture, this would render truncated ROCK entirely inactive! Also it is unclear why Rho plays a role in recruiting ROCK1 by binding to the

PHC1 domain but would then inhibit ROCK1 kinase activity. This behavior appears rather counterproductive. While the resolution of these key contradictions may be outside of the scope of the current manuscript, the authors should at least provide a new discussion of these results giving proper coverage of the prior research, which contradicts their conclusions.

Minor issues.

In current Discussion, the sentence "Intriguingly, based on the well-known heptad and hendecad repeat sequence motifs..." the authors introduce a very important structural hypothesis/ result that shows that ROCK1 molecule has a bend or kink at the RBD. This structural feature is important for membrane bound active Rho to bind to ROCK1 (and likely 2). As it stands, the sentence is cryptic, as very few people in the readership of JCB would know anything about heptad and hendecad structural features in CC domains. Also, here the authors should cite a recent structural biology paper (Hajdu et al., 2020 33247217) since it also introduces the concept of the CC domain being doubled over/ kinked in the vicinity of the RBD. Hajdu et al. offered their own structural model (see Fig. 3) to address the argument of Trubestein et al. 2015 regarding the impossibility of Rho binding to the RBD. Interestingly, the authors of Hejdu et al. posit that ROCK1 is a mixture of folded on itself and extended conformers, keeping the interaction between the PHC1 and the N-term of ROCK1 as an autoinhibitory interaction relieved by active Rho. The authors should substantially extend the discussion of the above kink proposal, discuss work of Hejdu et al. and even consider moving this section from the discussion to the Results as a plausible structure prediction.

The English language of the manuscript could be substantially improved. The reviewer found many unclear, poorly worded sentences, too many to list them here, that can be substantially improved by re-wording.

Response to reviewers' comments:

We thank the reviewers for their constructive criticism which have allowed us to revise and improve our manuscript. A point-by-point discussion is presented below. Our responses are highlighted in green:

Reviewer #1:

The manuscript by Gierse and colleagues employs an experimental model previously developed in their laboratory, which relies on overexpression of a microtubule-binding-deficient mutant of the Rho-GEF GEF-H1. This system generates cortical contraction pulses at the cell membrane. These pulses recruit active RhoA and ROCK1 to the plasma membrane through a signaling pathway previously described by the authors. This pathway combines a rapid positive feedback loop, mediated by the interaction between GEF-H1 and RhoA, with a slower negative feedback loop mediated by Myosin II.

In the present study, the authors propose that a new RhoA-binding site in ROCK1 facilitates its recruitment to the plasma membrane upon RhoA activation by GEF-H1. This site, located in the C-terminal PH domain, has not been previously identified. The authors further suggest that this newly described interaction contributes to downstream myosin activation by acting synergistically with the canonical RBD domain, positioning ROCK1 at the plasma membrane in a manner that promotes Myosin II activation.

The model system utilized allows for precise perturbations of the system, which the authors utilize to identify the domains in ROCK1 that mediate the recruitment. In addition, the use of an optogenetic system allows them to control the recruitment of ROCK1 constructs to the membrane in space and time.

Overall, the experiments are sound and thorough and systematically dissect the pathway. The results are novel and provocative, and may shed light on some previously contradictory or ambiguous findings.

We thank the reviewer for this very positive feedback.

However, there are also important concerns.

The main issue in this manuscript is that, even though this new RhoA binding site is proposed, is never directly validated. Recruitment of ROCK1 to the membrane mediated by this domain (PHC1), correlates with GEF-H1 mediated activation of RhoA, but the authors provide no evidence that this occurs through a direct interaction with RhoA. There is also no evidence that the interaction with Rho is nucleotide dependent. Without confirmatory biochemical or biophysical experiments, the author's conclusions, even though supported by the data, remain speculative.

There are several alternative explanations for the observed recruitment of ROCK1 via PHC1. The recruitment could still be Rho-GTP-dependent, as the data suggest, but not necessarily

mediated through direct RhoA binding. For example, PHC1 could interact with a Rho effector, with specific membrane lipids, or with GEF-H1 itself. Because this proposed interaction is central to the manuscript's conclusions, direct validation is essential. Without it, the authors would need to rewrite the manuscript, toning down the interpretation and significance of their findings to reflect this uncertainty. The absence of this evidence also decreases the significance of their findings.

In summary, this is an interesting and well-executed study that addresses an important and timely question in RhoA-ROCK signaling dynamics. However, the lack of direct biochemical evidence for the proposed RhoA-PHC1 interaction represents a major weakness.

We thank the reviewer for this important point. We agree that the original manuscript did not sufficiently distinguish between direct biochemical binding of active RhoA to the isolated ROCK1 PHC1 domain and the Rho-dependent PHC1 recruitment observed in cells.

To address this concern, we performed several additional experiments to test whether the PHC1 domain directly interacts with active RhoA. First, we purified the mPHC1 domain after exogenous expression with the aim to perform direct biochemical interaction assays. Unfortunately, the mPHC1 domain precipitated shortly after purification and could therefore not be used to evaluate a direct protein interaction. To enable a more native folding and post-translational context, we then performed pull-down experiments of tagged proteins in mammalian cells. Unfortunately, only unspecific interactions were detected, suggesting that the overexpressed mPHC1 domain may be prone to nonspecific interactions under these conditions.

We therefore agree with the reviewer that the current data does not support the conclusion that PHC1 is an autonomous RhoA-binding domain. We have revised the manuscript accordingly, including the title, abstract, results and discussion. Furthermore, we included the results of the pull down assay in HEK cells to document the experimental difficulties associated with this approach. We no longer present PHC1 as a direct RhoA-binding domain and have removed or qualified statements implying a direct binary PHC1–RhoA interaction. Instead, we now describe PHC1 as a domain that mediates residue-dependent recruitment to active Rho signaling zones in cells.

Importantly, the central cell biological conclusion remains supported by several independent observations: PHC1 recruitment closely follows GEF-H1/Rho activity in space and time, acute local recruitment of GEF-H1 is sufficient to induce local PHC1 recruitment, and mutation of defined PHC1 residues strongly reduces this response. These results argue against nonspecific membrane association as the sole explanation. However, because direct specific binding was not detected in the assays described above, we now explicitly discuss alternative mechanisms, including the possibility that PHC1-dependent recruitment requires membrane context, full-length ROCK1 architecture, cooperative engagement with the canonical RBD, local Rho density, avidity effects, or another Rho-dependent cortical factors such as other Rho effectors. We describe these observations in the Results section (page 11, para 2-3) to summarize the biochemical experiments and their interpretation.

Other concerns/comments.

-In most experiments, a line is drawn across the cell to generate kymographs, and a small Roi is used to measure the intensity changes of the different markers over time. Judging by the images provided, where these lines/ROI may affect the outcome of the measurements. How were these positions chosen for each experiment?

We chose the line regions for kymographs to convey a representative depiction of the corresponding signal dynamics. For almost all kymographs (except for Figure S1B) we also provide the corresponding video, which shows all dynamics in the cells shown in the figures. We would like to clarify that the choice of the line regions only affect the kymograph and do not affect the measurements of pulse height, pulse frequency, standard deviation, or perturbation or response signals, which were measured for statistical analyses.

The measurements of pulse height, pulse frequency and standard deviation were always based on a threshold-defined mask that covers the whole cell attachment area. Only the periphery of the attachement area was excluded from analysis (as detailed in Methods), to avoid a potnetial influence of cell protrusion/retraction dynamics on these measurements.

The measurements of perturbation or response signals were always perfomed at the optogenetic perturbation spot and were therefore also independent of the kymograph lines shown for these experiments.

We clarified this in the methods section “Image and video analysis”.

- The manuscript would benefit from additional information on the number of biological replicates and cells analyzed in each experiment. Ideally, individual experimental means should be shown (e.g., in Fig. 2E-H).

The number of cells analyzed are given in the figures. If different measurements were performed on a single set of experiments, this number was only given once (e.g. panels 2E-H all belong to the same set of cells with n>=27 cells). To increase clarity, we now report the number of analyzed cells in each figure panel. We agree that the data shown in Fig 2E-H is particularly important, as it demonstrates the highly reproducible recruitment of the PHC1 domain to Rho activity pulses, and we therefore now indicate the mean of each individual replicate in the scatterplots in these figure panels.

-Several movies are included in the supplemental material, but these are not referenced in the main text or figure legends. The reader has no way of knowing they exist.

We added links to all the movies in the revised manuscript.

-Details on statistical analyses are lacking. While some figures display p-values, there is no information on the type of test used in each case.

We added detailed information about all statistical tests in the figure legend and additionally stated that all tests were two-sided in the methods section.

- In Figure 5B (CA ROCK1), the example cell shown is of very poor quality (the accompanying movie as well). The signal from the expressed constructs appears much weaker than in other conditions (presumably this is among the better cells imaged). This raises concerns about differences in expression levels among constructs and how these might affect the results. There is no indication that the authors controlled for expression levels-by quantifying intensity, generating stable lines, or using Western blotting. This is a limitation, especially considering that some experiments analyze relatively few cells (e.g., as few as 14).

We agree with the reviewer that expression and recruitment levels are potentially important for the optogenetic recruitment experiments and that this particular cell showed weaker signals. We therefore replaced this CA-ROCK1 example with a cell that is more representative and comparable to the related conditions in terms of construct expression levels. Importantly, our quantitative analysis does not rely on visual comparison of representative images. For each cell, we quantified both the intensity of the recruited ROCK1 perturbation construct at the optogenetic recruitment site and the corresponding myosin response. These values are shown separately in Fig. 5E–F. This analysis shows that the reduced myosin responses cannot be explained simply by stronger recruitment of the wild-type construct or weaker recruitment of these mutants. We have clarified in the Methods that the perturbation intensity refers to the fluorescence intensity of the recruited ROCK1 construct at the perturbation site, whereas the response intensity refers to the mCherry–Myosin IIa signal measured at the same site and time window (see also last comment of this reviewer below).

Minor issues

-In p2, second paragraph (Introduction), I believe "Figure 4A" should be replaced by "Figure 1A"

Yes, we corrected this typo.

-In p11 last line, "Figure C-D" is missing the figure number

This should refer to Figure S4. This is now corrected.

-In p12 last paragraph, second line, I believe "mutAPH is the single mutant" and "mutBPH" is the triple mutant, not the other way round as stated.

Yes, we corrected this typo.

-in Figure 5E-F is not clearly what mean perturbation intensity and mean response mean and how it was measured. One can infer that one is the recruitment of each of the ROCK1 construct and the other was myosin recruitment, but details are insufficient.

We added additional details to the Methods section “Analysis of perturbation response kinetics” to clarify these measures.

Reviewer #2:

The manuscript by Gierse et al. provides interesting novel results and analysis, which both shed a new light on the enigmatic nature of ROCK1 biology and raise new questions that need to be addressed.

The authors start with the demonstration that ROCK1 and 2 are recruited to the pulses of actomyosin contraction induced by CA GEF-H1. They then proceed to show that all ROCK fragments previously described as ROCK domains interacting with active Rho are dispensable for the recruitment of ROCK1 into the pulses of contraction. The authors demonstrate that the C-terminal PH domain split by C1 domain (PHC1 in their nomenclature), in fact, interacts with active Rho providing two additional C-terminal membrane-binding sites to the ROCK1 kinase dimer. They further identify specific residues responsible for the binding of PHC1 and Rho and generate a mutant (mutC PH) that fails to interact with Rho. Interestingly, the authors find that the "classical" RBD proposed to interact with active Rho in the earlier literature, still plays that role and can be engaged by the membrane-bound Rho at the same time as the C-terminal PHC1 domains, despite being located 17nm away from the ROCK C-terminus along the dimeric CC domain. Satisfyingly, only a mutant in both PHC1 and RBD domains fails to be recruited to the contraction pulses.

These novel results are important and lay to rest some earlier controversies (Trubestein et al, 2015). An additional related result (see more below) is mentioned somewhat in passing, only in the Discussion section (should be Conclusion as the authors have main results labeled as Results and Discussion).

We thank the reviewer for this very positive feedback.

Major issue.

While the above results pertaining to the role of ROCK1 interaction with Rho for localization to the membrane appear to be clear and logical, the proposed by the authors role of Rho in the activation of ROCK1 opens new major controversies in the field.
In this section, the authors use their in-house-built optogenetics system to recruit various ROCK1 constructs. Optogenetic recruitment of FL ROCK1 resulting in myosin-2 activation suggests that active Rho is not essential for ROCK1 activation, although Fig. 5D might hint that the activity of the recruited kinase is not optimal.

We did not intend to convey that Rho is not essential for ROCK1 activation, but based on this data instead propose that recruitment of ROCK1 to the plasma membrane is sufficient for ROCK1 activation.

Then the authors show that ROCK1-delta PHC1 has no activity once recruited in the same fashion. Moreover, FL ROCK1 mutated in PHC1 binding to Rho (mutC PH) is 3 times more active

than WT ROCK1. These results are highly surprising in the light of earlier published results that show that the interaction of PHC1 domain and the N-term of ROCK1 is autoinhibitory and is relieved by binding to active Rho. The authors mention one original paper in conjunction with this result (Leung et al., 1996) and cite an obscure chapter (Khalil 2010), which this reviewer could not find on the internet. The authors are sent to more popular and easily accessible reviews (Julian and Olson, 2014 Pubmed 25010901; Hartmann et al., 2015 /26635606/) that introduce the primary literature supporting ROCK activation by Rho.

We changed the reference to these more accessible reviews.

Those results clash with the newly presented by the authors results on OG recruitment. For example, the results by Gierse et al. contradict a large body of evidence showing that, in apoptosis, caspases produce constitutively active ROCK1 and 2 by precisely removing the C-terminal PHC1 domain. In authors picture, this would render truncated ROCK entirely inactive! Also it is unclear why Rho plays a role in recruiting ROCK1 by binding to the PHC1 domain but would then inhibit ROCK1 kinase activity. This behavior appears rather counterproductive. While the resolution of these key contradictions may be outside of the scope of the current manuscript, the authors should at least provide a new discussion of these results giving proper coverage of the prior research, which contradicts their conclusions.

We thank the reviewer for this important criticism. We agree that the optogenetic recruitment experiments raise questions in light of previous models of ROCK regulation, and we have substantially expanded the Discussion to address this point.

We do not think that our data contradict caspase-mediated activation of ROCK1 or ROCK2. Instead, we now distinguish more clearly between global ROCK activation and local ROCK1-dependent signal transduction at the plasma membrane (Discussion, page 19, para 1). Caspase cleavage removes the C-terminal regulatory region and can generate globally active ROCK during apoptosis. Similarly, the constitutively active ROCK1 truncation used in our study, corresponding to ROCK1-Δ4 in Ishizaki et al., can induce global contractile phenotypes when overexpressed. However, our optogenetic assay addresses a different question: whether acute local recruitment of specific ROCK1 constructs to a defined plasma membrane region is sufficient to induce a local myosin response.

This distinction helps reconcile the apparently conflicting observations. The inability of locally recruited CA-ROCK1 to induce a detectable local myosin response does not mean that this construct lacks catalytic activity in cells. Rather, it suggests that local myosin activation at the plasma membrane requires appropriate molecular geometry, cortical positioning, substrate access, or regions of ROCK1 downstream of the kinase domain. Likewise, the strong reduction in myosin recruitment after PHC1 deletion and the enhanced late response of the PHC1 point mutant suggest that PHC1 has roles beyond simple autoinhibition.

We have also revised the Discussion to treat earlier autoinhibitory models more explicitly and more cautiously (Discussion, starting on page 19 para 2). We now state that our data do not exclude an interaction between the N- and C-terminal regions of ROCK. Instead, we propose that such regulation is likely context-dependent and that the C-terminal region may contribute to ROCK1 function through multiple mechanisms, including autoinhibition, membrane

recruitment, conformational positioning, and delayed regulation of local myosin activation .

Minor issues.

In current Discussion, the sentence "Intriguingly, based on the well-known heptad and hendecad repeat sequence motifs..." the authors introduce a very important structural hypothesis/ result that shows that ROCK1 molecule has a bend or kink at the RBD. This structural feature is important for membrane bound active Rho to bind to ROCK1 (and likely 2). As it stands, the sentence is cryptic, as very few people in the readership of JCB would know anything about heptad and hendecad structural features in CC domains. Also, here the authors should cite a recent structural biology paper (Hajdu et al., 2020 33247217) since it also introduces the concept of the CC domain being doubled over/ kinked in the vicinity of the RBD. Hajdu et al. offered their own structural model (see Fig. 3) to address the argument of Trubestein et al. 2015 regarding the impossibility of Rho binding to the RBD. Interestingly, the authors of Hejdu et al. posit that ROCK1 is a mixture of folded on itself and extended conformers, keeping the interaction between the PHC1 and the N-term of ROCK1 as an autoinhibitory interaction relieved by active Rho. The authors should substantially extend the discussion of the above kink proposal, discuss work of Hejdu et al. and even consider moving this section from the discussion to the Results as a plausible structure prediction.

We thank the reviewer for highlighting this important point. We agree that the coiled-coil kink/bend model was introduced too briefly in the original manuscript and was not accessible to a general JCB readership. We have therefore expanded this section considerably.

In the revised manuscript, we now explain why interruptions in the canonical coiled-coil pattern could generate a flexible bend near the canonical RBD (Discussion, page 16, para 2). We also cite and discuss the study by Hajdu et al., which proposed conformational rearrangements of ROCK and considered how more compact conformations could allow communication between membrane-proximal Rho binding and kinase regulation (Discussion, page 17, para 1). In addition, we discuss a recent preprint by Dubey et al. (https://doi.org/10.21203/rs.3.rs-8784410/v1), which reports structural evidence for an elongated ROCK1 coiled-coil and possible higher-order assembly through dimer–tetramer transitions (Discussion, page 17, para 1). We present these structural considerations as possible mechanisms that could explain how the RBD, PHC1 domain, and coiled-coil architecture cooperate in ROCK1 recruitment and local signal transduction.

The English language of the manuscript could be substantially improved. The reviewer found many unclear, poorly worded sentences, too many to list them here, that can be substantially improved by re-wording.

We carefully proof-read the manuiscript to identify and correct potentially unclear or poorly worded sentences.

August 25, 2026

RE: Life Science Alliance Manuscript #LSA-2026-03885-T

Dr. Leif Dehmelt
TU Dortmund University
Otto-Hahn-Straße 6
Dortmund 44227
Germany

Dear Dr. Dehmelt,

Thank you for transferring your manuscript entitled "The ROCK1 PHC1 domain mediates recruitment to Rho activity zones and contractility signaling". We apologise for the delay in communication due to editor availability issues.

In accordance with our communicated offer, we would be happy to publish your paper in Life Science Alliance pending resolution of the below mentioned points and final revisions necessary to meet our formatting guidelines:

- Discuss possible hypotheses to explain confounding results around role of active RhoA in ROCK activation (Reviewer 2 - third paragraph)
- Temper claims related to direct (PHC1domain-active RhoA) interaction throughout the ms (Reviewer 1 - Figure 5, 6, Alphafold). Related to this, expand on your explanation on page 15 that PHC1 domain mediates context-dependent recruitment to cortical Rho activity zones.
- Provide representative images for altered phalloidin staining (Figure 5G, Reviewer 1).

Please include a point-by-point response and highlight any changes in the manuscript.

MANUSCRIPT ORGANIZATION AND FORMATTING:

To avoid unnecessary delays in the acceptance and publication of your paper, please read the following information carefully. Full guidelines are available on our Instructions for Authors page, https://www.life-science-alliance.org/authors

Submission of a paper that does not conform to Life Science Alliance guidelines will delay the acceptance of your manuscript.

-Please incorporate any description/information on methods from the supplemental section into materials and methods section in the main text. Accordingly remove the paragraph called "Supplementary Materials Summary".
-Please include molecular weight markers in Figure 2D.
-Kindly modify your statement on data availability in alignment with LSA's guidelines (https://www.life-science-alliance.org/editorial-policies#data-sharing).
-Please rename "Results and Discussion" to Results, since you have a separate discussion section.
-Please add ORCID ID for corresponding author--you should have received instructions on how to do so.
-Please add a Summary Blurb/Alternate Abstract in our system.
-Please add a Category for your manuscript in our system.
-Please add the X and Bluesky handles of your host institute/organization, as well as your own, and/or one of the authors, in our system.
-Please add authors' contributions to our system as well.
-Please use the [10 author names et al.] format in your references (i.e., limit the author names to the first 10).
-Please add a Conflict of Interest statement to your main manuscript text.
-There is a call-out for Fig. S2E, and this figure doesn't have this panel. Please correct.
-Please add callouts for Figures 5G; S1A-C and S3A-E to your main manuscript text.
-Please be sure that the authorship listing and order is correct.

We encourage our authors to provide original source data, particularly uncropped/-processed electrophoretic blots and spreadsheets for the main figures of the manuscript. If you would like to add source data, we would welcome one PDF/Excel-file per figure for this information. These files will be linked as supplementary "Source Data" files.

It is Life Science Alliance policy that if requested, original data images must be made available to the editors. Failure to provide original images upon request will result in unavoidable delays in publication. Please ensure that you have access to all original data images prior to final submission.

If you are planning a press release on your work, please inform us immediately to allow informing our production team and scheduling a release date.

We welcome submissions of potential cover images for the issue of LSA in which your work would appear. If you have high quality images associated with this work, please feel free to email these, with a caption, to the journal office.

LSA encourages authors to provide a 30-60 second video where the study is briefly explained. These videos will be appear embedded with the manuscript online at Life Science Alliance, and on social media to promote the published paper and authors (for examples, see https://docs.google.com/document/d/1-UWCfbE4pGcDdcgzcmiuJl2XMBJnxKYeqRvLLrLSo8s/edit?usp=sharing). Corresponding or first-authors are welcome to submit the video. Please submit only one video per manuscript. The video can be emailed to contact@life-science-alliance.org

FINAL FILES:

To upload the final version of your manuscript, please log in to your account: https://lsa.msubmit.net/cgi-bin/main.plex
You will be guided to complete the submission of your revised manuscript and to fill in all necessary information. Please get in touch in case you do not know or remember your login name.

The following items are required for acceptance.

-- An editable version of the final text (.DOC or .DOCX) is needed for copyediting (no PDFs).

-- High-resolution figure, supplementary figure and video files uploaded as individual files: See our detailed guidelines for preparing your production-ready images, https://www.life-science-alliance.org/authors

-- Summary blurb (enter in submission system): A short text summarizing in a single sentence the study (max. 200 characters including spaces). This text is used in conjunction with the titles of papers, hence should be informative and complementary to the title. It should describe the context and significance of the findings for a general readership; it should be written in the present tense and refer to the work in the third person. Author names should not be mentioned.

The license to publish form must be signed before your manuscript can be sent to production. A link to the license to publish form will be available to the corresponding author only. Please take a moment to check your funder requirements.

Reviews, decision letters, and point-by-point responses associated with peer-review at Life Science Alliance will be published, alongside the manuscript. If you do want to opt out of having the reviewer reports and your point-by-point responses displayed, please let us know immediately.

Thank you for your attention to these final processing requirements. Please revise and format the manuscript and upload materials as soon as you are able.

Thank you for this interesting contribution to the literature. We look forward to publishing your paper in Life Science Alliance.

Sincerely,

Sarita Hebbar, PhD
Scientific Editor
Life Science Alliance
http://www.lsajournal.org

Response to reviewers' comments:

We thank the reviewers for their constructive criticism and the editors for their clear guidelines for revision to improve our manuscript. A point-by-point discussion is presented below. Our responses are highlighted in green:

Editorial revision requests:

- Temper claims related to direct (PHC1 domain-active RhoA) interaction throughout the ms (Reviewer 1 - Figure 5, 6, Alphafold).

We introduced several changes to address this point. We also agree with the assessment of reviewer #1 that the previous manuscript was convoluted, and we therefore aimed to temper our claims, and at the same time we aimed to clarify both the limitations and the value of the Alphafold-based predictions. To do this we made the following edits:

1. We included several minor edits to improve clarity, in particular concerning wording that would suggest a direct interaction between PHC1 and RhoA thoughout the manuscript.

2. The Alphafold predictions are now framed in a different context that better aligns with the experiental observations. The primary result of the Alphafold-based predictions is that several different small GTPases, including several members of the Rho subfamily such as RhoA, B, C as well as Cdc42,Rac1, RhoG, were predicted to be potential interaction partnerns of the PHC1 domain. The previous Figure S2B supports this idea and we now shifted this panel to the beginning of the main Figure 3. The predicted complex with RhoA is shown as a representative example for the various potential interaction partners including the Rho family small GTPases and related molecules. To avoid potential misinterpretatons, we also removed the explicit RhoA label in Figure 3B and 3C and now only refer to a "small GTPase" in this context. (Page 8, para 3 – Page 9, para 2)

3. We clarified our interpretation related to the pull-down assay: We now state that this assay "did not provide evidence for a direct high-affinity PHC1-RhoA- interaction, but it does not exclude interactions with other Rho-family GTPases like the highly related RhoB and RhoC isoforms, or a weaker interaction with RhoA that could be strengthened by cooperative effects at the plasma membrane that might depend on GTPase membrane anchoring, protein-lipid interactions or additional cellular co-factors that are absent in the pulldown conditions." (Page 11, para 4).

4. Based on these clarifications we then rewrote the first paragraph of the discussion to make a case for an interaction between the PHC1 domain binding to a Rho family small GTPase as the "simplest explanation that is consistent with our combined computational and cellular evidence." (Page 15, para 4) We make this argument based on three converging observations that support this idea: 1. The recruitment of the PHC1 domain to Rho activity zones. 2. The higher confidence in the predictions for several Rho-family GTPases compared to control complexes containing Ras-, Rap- and Rab-family GTPases. 3. The disruption of PHC1 domain targeting to Rho activity zones by mutations predicted to disrupt the putative binding interface. Importantly, we explicitly state this as a leading hypothesis and provide additonal,

alternative explanations, and discuss these alternative explanations in the context of the experimental evidence. We believe that these interpretations improve the logic and readability of the manuscript and clearly deliniate which statements are supported by the experimental evidence and which are hypothetical speculation.

Related to this, expand on your explanation on page 15 that PHC1 domain mediates context-dependent recruitment to cortical Rho activity zones.

5. In our revised mansucript, we now avoid the ambiguous phrase “context-dependent” and instead describe the local membrane context, in particular negative charges at the plasma membrane and corresponding positive charges in several regions of the ROCK1 C-terminus as a potentially important factor for effective plasma membrane recrutiment (Page 11, para 4; Page 15, para 4).

- Discuss possible hypotheses to explain confounding results around role of active RhoA in ROCK activation (Reviewer 2 - third paragraph)

In the third paragraph, Reviewer #2 remarks that we “…did not provide any new experimental clarifications or plausible hypotheses explaining the conundrum…” about puzzling results concerning the functional role of the PHC1 domain. To address this concern, we significantly rewrote the discussion section "Potential role of the PHC1 domain in transduction of upstream signals to Myosin activation":

7. The first puzzling result is that artificial targeting of full length ROCK1 leads to a strong myosin response, while artificial targeting of full length ROCK1 lacking the PHC1 domain has only very little effect. Regarding this point we clearly state that "plasma membrane targeting alone via an artificial domain cannot substitute all critical functions of the PHC1 domain..." and we argue that an intact PHC1 domain is important for correct and stable positioning of ROCK1 at the cell cortex (Page 18, para 2 – Page 19, para 1). We believe that this is a relatively strightforward explanation. As we do not have further structural data, we would like to avoid being overly speculative.

8. To address the second puzzling result, that "PHC1 mutations in full length ROCK1 that reduce recruitment to Rho activity zones nevertheless increased the Myosin response after optogenetic plasma membrane targeting" (Page 19, para 1), we simlified and extended our interpretation. First, we removed statements about negative feedback to avoid overinterpretation, but instead just interpret this effect as a delayed inhibitory activity (Page 19, para 1). In order to keep our interpretations close to experimental evidence, we first propose a mechanism based on a competitive association of the PHC1 domain with a Rho family GTPase (according to the justification detailed above in points 2, 3 and 4). We explicitly refer to this proposed mechanism as one possibility (Page 19, para 4). We then provide an alternative interpretation based on the idea that a different GTPase might bind and discuss potential candidates that could indeed have an inhibitory activity towards myosin, such as Rnd3 (Page 19, para 5). We then provide a third possibility, that this effect might be due to interaction with another protein or protein domain, for example the ROCK N-terminus via an intramolecular interaction and discuss this in the context of previous classical models of ROCK regulation (Page 19, para 5 – Page 20, para 1). We believe that these hypotheses provide

several reasonable explanations for this observation and we discuss these hypotheses in the following paragraphs (Page 20, para 2- para 3).

- Provide representative images for altered phalloidin staining (Figure 5G, Reviewer 1).

We now provide these images in Figure S3.

September 16, 2026

RE: Life Science Alliance Manuscript #LSA-2026-03885-TR

Dr. Leif Dehmelt
TU Dortmund University
Otto-Hahn-Straße 6
Dortmund 44227
Germany

Dear Dr. Dehmelt,

Thank you for submitting your Research Article entitled "The ROCK1 PHC1 domain mediates recruitment to Rho activity zones and contractility signaling". It is a pleasure to let you know that your manuscript is now accepted for publication in Life Science Alliance. Congratulations on this interesting work.

The final published version of your manuscript will be deposited by us to PubMed Central upon publication.

Your manuscript will now progress through copyediting and proofing. At the proofing stage, we recommend that you correct the sub-panel label for Figure S3F (for representative images of U2OS cells labelled with phalloidin).

It is journal policy that authors provide original data upon request.

Your article will publish open access upon publication under a CC-BY license.

Reviews, decision letters, and point-by-point responses associated with peer-review at Life Science Alliance will be published, alongside the manuscript. If you do want to opt out of having the reviewer reports and your point-by-point responses displayed, please let us know immediately.

IMPORTANT: If you will be unreachable at any time, please provide us with the email address of an alternate author. Failure to respond to routine queries may lead to unavoidable delays in publication.

Scheduling details will be available from our production department. You will receive proofs shortly before the publication date. Only essential corrections can be made at the proof stage so if there are any minor final changes you wish to make to the manuscript, please let the journal office know now.

DISTRIBUTION OF MATERIALS:
Authors are required to distribute freely any materials used in experiments published in Life Science Alliance. Authors are encouraged to deposit materials used in their studies to the appropriate repositories for distribution to researchers.

You can contact the journal office with any questions, contact@life-science-alliance.org

Again, congratulations on a very nice paper. I hope you found the review process to be constructive and are pleased with how the manuscript was handled editorially. We look forward to future exciting submissions from your lab.

Sincerely,

Sarita Hebbar, PhD
Scientific Editor
Life Science Alliance
http://www.lsajournal.org
